# Lithium‐Doped Hydroxyapatite Nanowires Rejuvenate Senescent Macrophages Via the Lysosome‐Dependent Ferroptosis Axis to Promote Aged Bone Regeneration

**DOI:** 10.1002/advs.78424

**Published:** 2026-10-25

**Authors:** Baixi Chen, Ziduan Ding, Ruomei Li, Cheng Zhu, Zhiyu Zhu, Zitong Yang, Luyao Qu, Jin Hao, Lunguo Xia, Bing Fang, Lu Liu

**Affiliations:** ^1^ Department of Orthodontics Shanghai Ninth People's Hospital Shanghai Jiao Tong University School of Medicine Shanghai Jiao Tong University Shanghai China; ^2^ National Clinical Research Center for Oral Diseases Shanghai Key Laboratory of Stomatology and Shanghai Research Institute of Stomatology Shanghai China; ^3^ Laboratory of Oral Microbiota and Systemic Diseases Shanghai Ninth People's Hospital Shanghai Jiao Tong University School of Medicine Shanghai China

**Keywords:** ferroptosis, hydroxyapatite, lithium, lysosomal homeostasis, macrophages, senescent bone repair

## Abstract

Senescent bone repair remains a formidable clinical challenge without an entirely satisfactory strategy. Emerging evidence has identified macrophage senescence as a critical determinant of impaired bone healing in the aged skeleton, and precisely reversing macrophage senescence has therefore emerged as a highly promising therapeutic approach. In this study, guided by multi‐omics features of senescent macrophages, we develop lithium‐doped hydroxyapatite nanowires (Li‐nHAP) and demonstrate that Li‐nHAP effectively alleviates macrophage senescence and subsequently enhances the osteogenic differentiation of aged bone marrow mesenchymal stem cells. Mechanistically, we identify the lysosome‐dependent ferroptosis axis as a previously unrecognized target mediating the anti‐senescent effects of Li‐nHAP in bone marrow‐derived macrophages. Specifically, Li‐nHAP re‐establishes lysosomal homeostasis, including restoring acidity, preserving membrane integrity, maintaining iron storage, and enhancing hydrolytic activity, thereby suppressing ferroptosis and ultimately reversing macrophage senescence. In vivo studies further validate that Li‐nHAP significantly alleviates macrophage ferroptosis and senescence, reduces local iron accumulation, and accelerates bone regeneration in an aged mandibular defect model. Collectively, these findings establish Li‐nHAP as a novel osteo‐immunomodulatory biomaterial that offers a promising strategy for the repair of age‐related bone defects.

## Graphical abstract

Li‐nHAP+GelMA promotes mandibular bone regeneration in aged mice by remodeling the senescent immune microenvironment. Local delivery of Li‐nHAP restores lysosomal homeostasis in senescent macrophages, reduces intracellular labile Fe^2^^+^ accumulation, ROS production, and lipid peroxidation, thereby suppressing macrophage ferroptosis and senescence‐associated SASP signaling. This macrophage rejuvenation alleviates the aged inflammatory microenvironment and enhances osteogenesis, ultimately improving bone repair in mandibular defects.

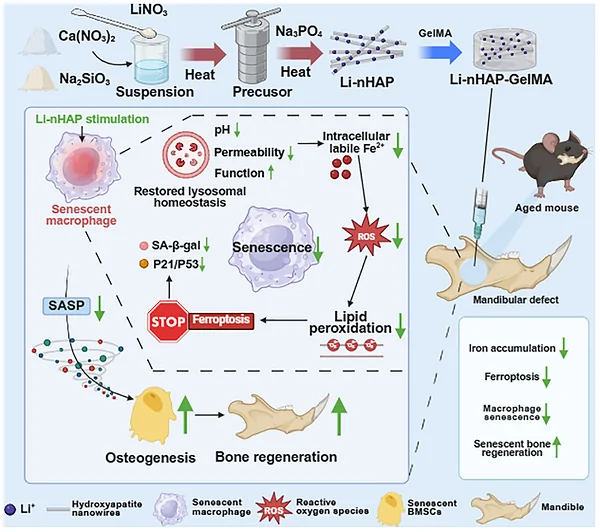

## 1 Introduction

Driven by the rapid aging of the global population, the incidence of extensive bone trauma, fractures, and bone defects has surged among the elderly. Bone repair in aged individuals remains a formidable clinical challenge, frequently leading to diminished quality of life and even reduced lifespan. While these age‐related regenerative deficits have traditionally been attributed to the functional exhaustion and diminished osteogenic capacity of bone marrow mesenchymal stem cells (BMSCs), emerging evidence emphasizes that aging of the immune system is another critical determinant of impaired skeletal regeneration [1, 2]. Notably, the proportion of macrophages within the bone marrow increases significantly with advancing age. These cells exhibit a distinct pro‐inflammatory and senescent profile, contributing to systemic aging and tissue dysfunction [3, 4]. Recent studies further demonstrated that the accumulation of senescent phagocytic cells within the aged bone marrow impairs the osteogenesis of BMSCs and impedes fracture healing through senescence‐associated secretory phenotype (SASP)‐mediated paracrine signaling [5]. Consequently, bone marrow‐derived macrophages (BMDMs) are not merely passive bystanders in the aging process, but rather function as active propagators. Current strategies targeting senescent macrophages largely focus on identifying and neutralizing individual downstream SASP factors. However, the complexity and functional redundancy of the SASP network may limit the efficacy of single factor blockade [6]. Accordingly, elucidating the upstream mechanisms of macrophage senescence and designing targeted interventions represent a promising strategy for aged mandibular defect repair [7, 8].

Ferroptosis is an iron‐dependent, lipid peroxidation‐driven form of regulated cell death involved in various aging‐related diseases, including neurodegeneration and organ fibrosis [9, 10]. During natural aging, abnormal iron accumulation across tissues actively catalyzes oxidative damage and accelerates senescence, a pathological interplay recently conceptualized as “ferro‐aging” [11]. Notably, inhibiting this iron‐dependent cell death or the ‘ferro‐aging’ process contributes to the maintenance of organ function, the mitigation of senescence, and the extension of lifespan across multiple species [11, 12]. Importantly, ferroptosis has emerged as a key regulator of macrophage function and inflammatory status. Conversely, macrophage senescence has been shown to be accompanied by iron dyshomeostasis and heightened ferroptosis susceptibility [13, 14]. Reducing iron accumulation is recognized to restore metabolic homeostasis and preserve bone mass in aged bone [15]. However, within bone repair in aged individuals, the precise role and regulatory mechanisms of ferroptosis in macrophage senescence remain largely unexplored.

Lysosomes have been regarded as the central hubs for cellular iron storage and metabolism, and the disruption of iron homeostasis within these organelles is associated with various cancers and degenerative diseases [16, 17]. Accumulating evidence has identified these acidic organelles as initiators of iron‐dependent cell death, establishing their internal iron pools as reliable regulatory targets [18, 19]. Mechanistically, lysosomal dysfunction promotes cytoplasmic iron leakage, triggering Fenton reactions and lipid peroxidation—a hallmark of ferroptosis [20, 21]. Additionally, senescent cells exhibit enlarged and alkalinized lysosomes with reduced hydrolytic activity, which compromise autophagic clearance and accelerate the accumulation of damaged organelles [22]. In macrophages, lysosomal dysfunction impairs their phagocytic capacity and promotes the secretion of SASP factors, thereby contributing to age‐related tissue dysfunction [23]. More importantly, rescuing lysosomal impairments has been shown to effectively restore iron homeostasis and mitigate ferroptosis, thereby promoting post‐irradiation bone regeneration [24]. Despite these insights, whether lysosomal homeostasis is altered in macrophages within the aged bone microenvironment, and how lysosomal dysfunction regulates ferroptosis and macrophage senescence remain largely unexplored.

Given that the accumulation of senescent macrophages and SASP factors compromises regenerative outcomes, advanced biomaterials have emerged as a pivotal platform for anti‐senescence interventions [25, 26]. To identify the molecular features of senescent macrophages, we performed multi‐omics profiling, which revealed dysregulated ferroptosis and lysosomal dysfunction as two prominent signatures. These findings prompted us to design a biomaterial capable of targeting these pathways. Nanohydroxyapatite (nHAP) has been extensively employed as a biomaterial scaffold for promoting bone regeneration, owing to its excellent biocompatibility and osteoconductive properties [27, 28]. However, within the senescent microenvironment, unmodified nHAP struggles to achieve optimal therapeutic outcomes, largely due to its limited immunomodulatory capabilities. To overcome this limitation, doping nHAP with bioactive inorganic ions has emerged as a powerful strategy to impart anti‐senescence functions. Among candidate ions, lithium ions (Li^+^) are particularly attractive. Low‐dose lithium possesses broad anti‐aging potential [29], effectively alleviating cellular senescence [30] and reducing the risk of age‐related pathologies such as osteoporosis [31], sarcopenia [32] and oxidative stress [33]. Simultaneously, Li^+^ can modulate macrophage polarization, thereby inhibiting inflammation and promoting bone regeneration [34]. Moreover, lithium has been reported to regulate ferroptosis and to inhibit glycogen synthase kinase‐3β (GSK‐3β) [35], whose inhibition promotes the nuclear translocation of transcription factor EB (TFEB)—a master regulator of lysosomal biogenesis [36]. Given these promising attributes, we reasoned that incorporating lithium into nHAP could target ferroptosis and lysosomal dysfunction in senescent macrophages. However, whether lithium‐doped nHAP can alleviate macrophage senescence and promote aged bone regeneration needs to be systematically explored.

In this study, we first validated the pivotal regulatory role of macrophage senescence in bone regeneration, and then performed a multi‐omics analysis to characterize the distinct features of senescent macrophages. Building upon these insights, we purposefully engineered lithium‐doped hydroxyapatite nanowires (Li‐nHAP) with varying doping ratios and systematically evaluated their capacity to rejuvenate senescent macrophages and facilitate bone regeneration in aged mice. Our results demonstrated that Li‐nHAP effectively alleviates macrophage senescence by restoring lysosomal homeostasis and subsequently blocking the lysosome‐dependent ferroptosis cascade. To further assess its therapeutic potential in vivo, Li‐nHAP was incorporated into gelatin methacryloyl (GelMA) hydrogel for local delivery. Utilizing a critical‐size mandibular defect model in aged mice, we confirmed that Li‐nHAP significantly alleviates macrophage ferroptosis and senescence, reduces local iron accumulation, and accelerates bone repair. Collectively, our findings identify Li‐nHAP as a promising biomaterial strategy for age‐related bone defects, offering a novel approach that targets the lysosome–dependent ferroptosis axis to counteract macrophage senescence in bone repair.

## 2 Results and Discussion

### 2.1 Sn‐BMDMs Impair Osteogenesis of BMSCs and Exhibit Distinct Pro‐Senescent Profiles

Emerging evidence has established macrophage senescence as a driver of systemic aging, contributing to homeostatic disruption [3], hepatic metabolic dysfunction [4] and cognitive decline [37]. In the skeletal system, several studies have also implicated macrophage senescence in impaired fracture healing and age‐related bone repair deficits [5, 6]. However, the direct causal relationship between macrophage senescence and bone defect repair remains incompletely understood. To investigate whether macrophage senescence directly affects osteogenic differentiation, we treated young or senescent BMSCs with conditioned medium (CM) derived from young bone marrow‐derived macrophages (Yg‐BMDMs), senescent bone marrow‐derived macrophages (Sn‐BMDMs), or Sn‐BMDMs treated with the senolytic cocktail, dasatinib and quercetin (D&Q) [38, 39]. Compared with Yg‐BMDMs CM, Sn‐BMDMs CM significantly suppressed the osteogenic capacity of young BMSCs. Notably, this inhibitory effect was largely reversed when BMSCs were cultured with CM from D&Q‐treated Sn‐BMDMs (Figure S1A–E). More importantly, although senescent BMSCs exhibited inherently weaker osteogenic potential than young BMSCs, they responded similarly to the macrophage‐derived paracrine signals. Sn‐BMDMs CM further suppressed their osteogenic capacity, while CM from D&Q‐treated Sn‐BMDMs partially restored it (Figure S1F‐J). Collectively, these results demonstrated that Sn‐BMDMs critically impair the osteogenic differentiation of BMSCs, and that targeting macrophage senescence represents a viable strategy to counteract age‐related bone repair deficits.

To further dissect the molecular underpinnings of macrophage senescence, we performed multi‐omics analyses (transcriptomics, proteomics, and untargeted metabolomics) on Yg‐BMDMs and Sn‐BMDMs (Figure 1A). Principal component analysis (PCA) confirmed clear separation between the two groups at both the transcriptional and proteomic levels (Figure S2A,C). Differential expression analysis showed 4456 upregulated and 2681 downregulated genes, as well as 655 upregulated and 146 downregulated proteins in Sn‐BMDMs (Figure S2B,D). Gene Ontology (GO) enrichment analysis of the transcriptome revealed that differentially expressed genes were mainly enriched in inflammation and senescence related pathways (Figure 1B), consistent with the well‐established concept that senescent cells display a pro‐inflammatory phenotype [40]. GO and Kyoto Encyclopedia of Genes and Genomes (KEGG) enrichment analyses of the proteome further identified significant enrichment of lysosome‐related pathways, iron metabolism‐related pathways, and the peroxisome proliferator‐activated receptor (PPAR) pathway (Figure 1C,D). We next explored metabolic differences using untargeted metabolomics. Partial least squares‐discriminant analysis (PLS‐DA) revealed distinct metabolic profiles between Yg‐BMDMs and Sn‐BMDMs (Figure S2E), with 103 upregulated and 113 downregulated metabolites in Sn‐BMDMs (Figure S2F). KEGG enrichment analysis of the metabolome also highlighted lysosome and ferroptosis pathways (Figure 1E). Consistently, Gene Set Enrichment Analysis (GSEA) confirmed the upregulation of ferroptosis (Figure 1F), oxidative stress (Figure 1G), and inflammatory response (Figure 1H,I) during macrophage senescence, further validating the molecular signatures observed in earlier studies [4, 7]. Heatmaps of inflammation and ferroptosis‐associated genes further corroborated these findings (Figure 1J). Proteomic analysis confirmed increased expression of ferroptosis‐related proteins and decreased expression of lysosome‐related proteins in Sn‐BMDMs (Figure 1K). Taken together, our multi‐omics analyses suggest that dysregulated ferroptosis, lysosomal dysfunction, and enhanced inflammatory signaling are key features of Sn‐BMDMs. To further validate these observations in bone injury microenvironment, we re‐analyzed a public single‐cell RNA sequencing (scRNA‐seq) dataset (GSE280914) of fracture tissues from young and aged mice (Figure 1L) [41]. This dataset was selected because it provides periosteal immune‐cell scRNA‐seq profiles from both age groups in an independent fracture model, allowing us to directly test whether our identified macrophage signatures are recapitulated in the aged bone‐injury microenvironment. Macrophages in aged fracture tissues showed significantly higher senescence scores (Figure 1M), together with elevated SASP‐related gene expression and enhanced ferroptosis signatures (Figure 1N). Thus, the pro‐senescent and pro‐inflammatory profiles of aged macrophages, along with their activated ferroptosis and lysosomal dysfunction, are not merely in vitro phenomena but are also operative in the aged bone injury microenvironment. Therefore, engineering an advanced biomaterial platform capable of mitigating inflammatory response [42, 43], blocking ferroptosis [44, 45], and concurrently restoring lysosomal dysfunction [46, 47] represents a highly promising strategy to restore the deteriorated aged bone repair microenvironment.

**FIGURE 1:**
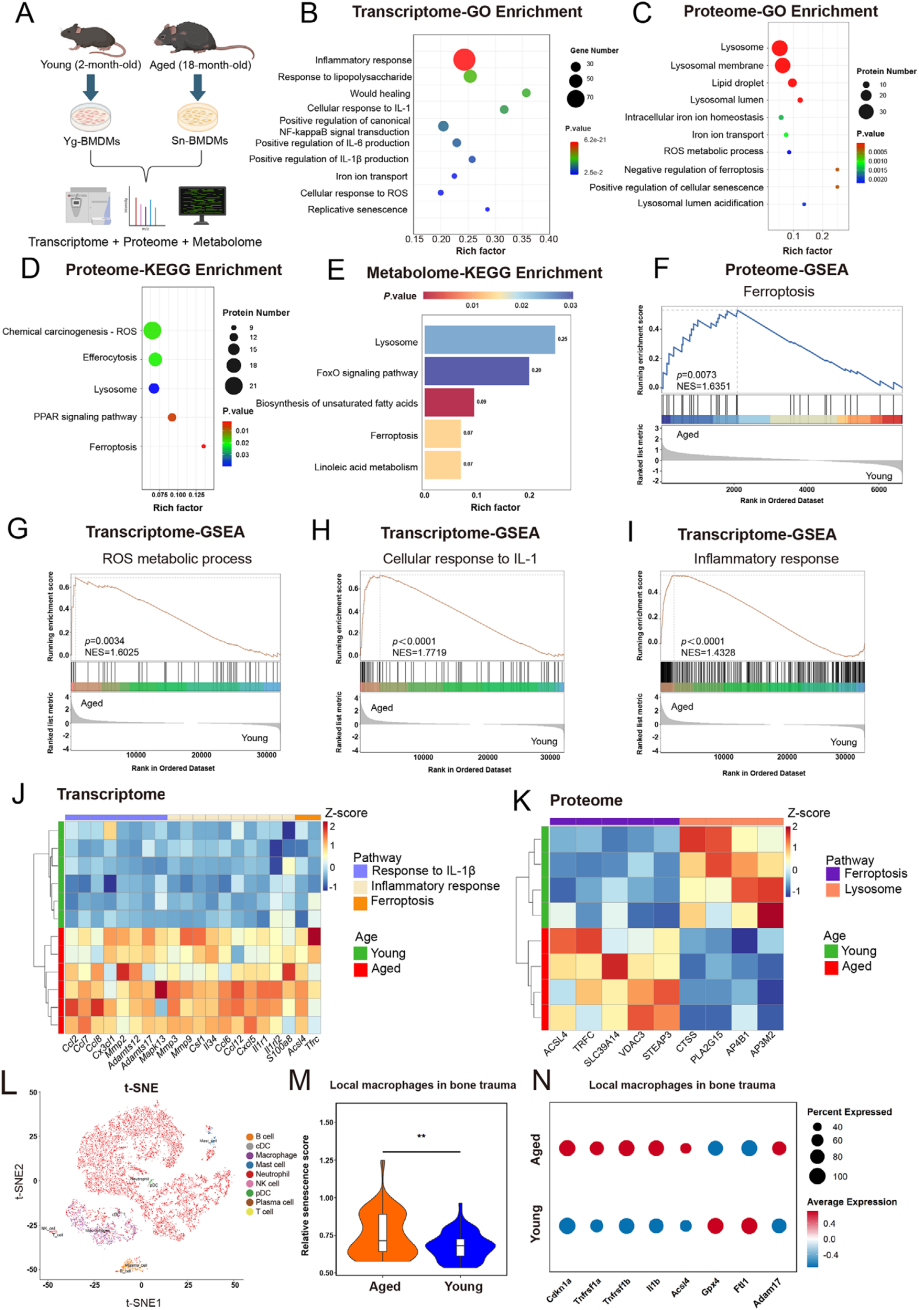
Multi‐omics integration reveals senescence phenotype, enhanced ferroptosis and lysosomal dysfunction in senescent macrophages. (A) Schematic diagram of the experimental design for multi‐omics analysis. Transcriptomic and metabolomic analysis (n = 6). Proteomic analysis (n = 4). (B–E) GO and KEGG enrichment analyses based on transcriptomics (B), proteomics (C, D), and untargeted metabolomics (E). (F–I) GSEA of ferroptosis (F), reactive oxygen species (ROS) metabolic process (G) and inflammatory responses (H, I). (J, K) Heatmaps of the differential expression of genes (J) and proteins (K) associated with inflammation, ferroptosis, and lysosomal function. (L–N) Re‐analysis of the public scRNA‐seq dataset (GSE280914) showing the t‐SNE plot (L), senescence score quantification (M), and expression profiles of senescence‐, SASP‐ and ferroptosis‐related genes (N) in local macrophages.

### 2.2 Characteristics of Lithium‐Doped Hydroxyapatite Nanowires

Given the reported roles of lithium in regulating inflammation, ferroptosis, and lysosomal function [48, 49, 50], we reasoned that incorporating lithium into hydroxyapatite nanowires (nHAP) might be effective to rejuvenate the aged bone microenvironment. Accordingly, we synthesized nHAP with different lithium doping ratios (0%, 5%, and 10%) via a hydrothermal process (Figure 2A). Scanning electron microscopy (SEM) and transmission electron microscopy (TEM) revealed the uniform short‐wire morphology for all products (Figure 2B–D), with an average length of approximately 210 nm (Figure 2E; nHAP, 205.6 ± 23.43 nm; 5%Li‐nHAP, 215.2 ± 20.38 nm; 10%Li‐nHAP, 207.8 ± 24.46 nm) and the average diameter of approximately 18 nm (Figure 2F; nHAP, 18.61 ± 1.19 nm; 5%Li‐nHAP, 18.43 ± 1.21 nm; 10%Li‐nHAP, 17.98 ± 1.66 nm), confirming that lithium doping did not significantly alter the geometric features of the nanowires. This nanoscale architecture resembles the collagen‐mineral organization of native bone, supporting Li‐nHAP as a biomimetic scaffold for cell adhesion and bone regeneration [51]. Time‐of‐flight secondary ion mass spectrometry (TOF‐SIMS) verified the presence and relatively uniform spatial distribution of lithium signals in Li‐nHAP (Figure 2G). The dynamic lithium release of Li‐nHAP was evaluated using inductively coupled plasma optical emission spectrometry (ICP‐OES) (Figure 2H). After incubation in phosphate‐buffered saline (PBS) at 37°C with gentle agitation, the released Li/(Li + Ca) ratio in PBS of 5%Li‐nHAP increased continuously from 0.037 to 0.187, reaching a plateau around day 21; whereas 10%Li‐nHAP exhibited a sustained release, with the ratio rising from 0.059 to nearly 0.291 by day 28. Fourier‐transform infrared spectroscopy (FTIR) revealed characteristic bands of nHAP, including the –OH stretching peak at 3558 cm^−^^1^, the PO_4_^3^^−^ vibration at 1024 cm^−^^1^, and phosphate bending bands at 603 cm^−^^1^ and 569 cm^−^^1^ (Figure 2I). X‐ray diffraction (XRD) patterns of the Li‐nHAP products were consistent with those of pristine hydroxyapatite at all doping ratios (PDF#74‐0565), indicating that lithium doping did not disrupt the crystal structure (Figure 2J). Brunauer–Emmett–Teller (BET) analysis showed that 5%Li‐nHAP possessed a high nitrogen adsorption capacity with pore size distributions showing mesopores at 3–4 nm and larger pores at 10–40 nm (Figure 2K). The smaller mesopores may facilitate tissue fluid infiltration, nutrient transport, and metabolic waste removal, whereas the larger mesopores may enhance protein adsorption and improve cell‐material interactions [52]. To further evaluate the stability of these nanowires, pH‐dependent dissolution behavior was examined under conditions spanning acidic to near‐physiological environments. All three nanowires exhibited greater dissolution under acidic conditions, with dissolution progressively decreasing as the pH approached neutrality (Figure 2L).

**FIGURE 2:**
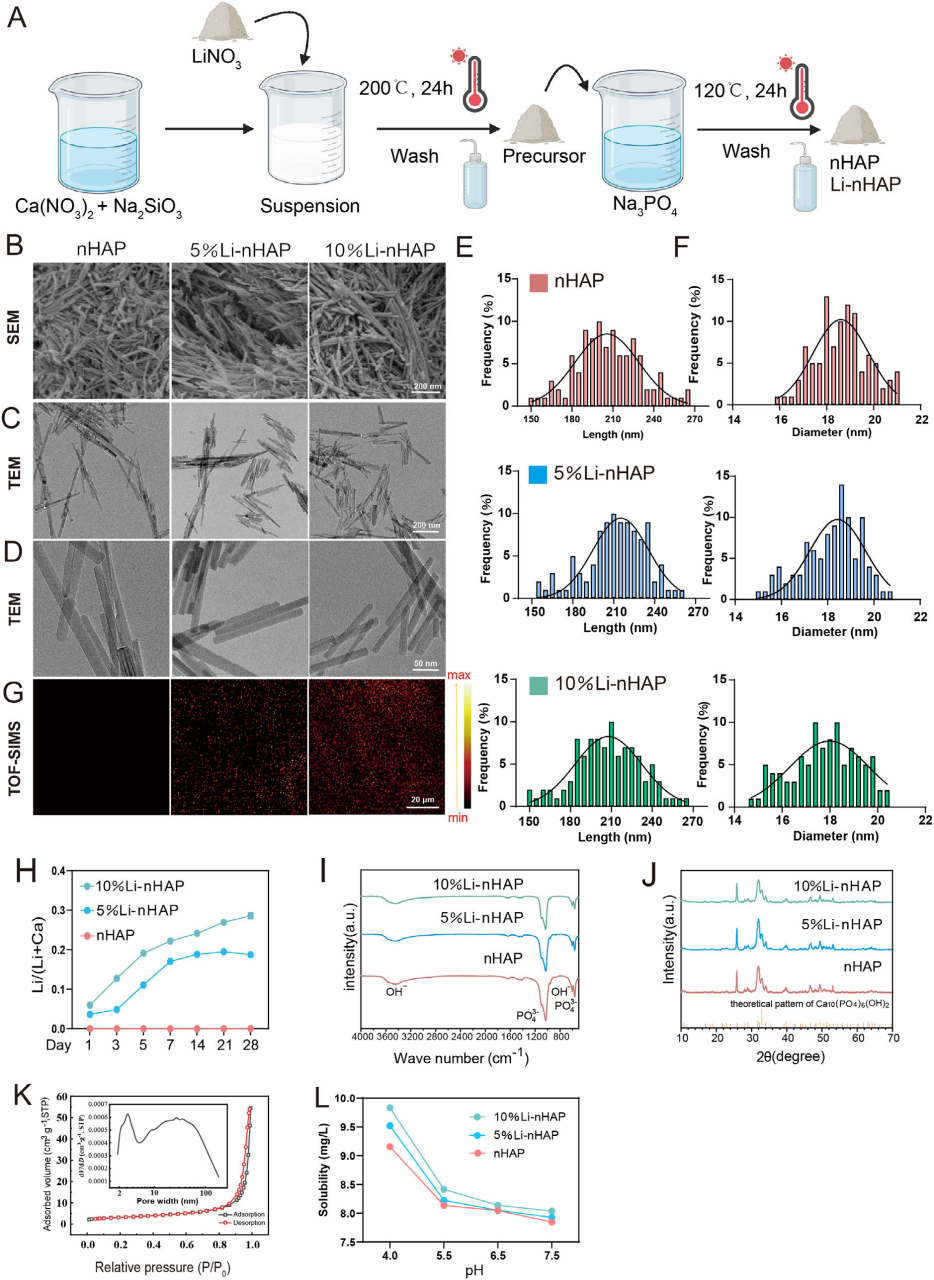
Synthesis and characterization of lithium‐doped hydroxyapatite nanowires. (A) Schematic illustration of the material synthesis process. (B) SEM images and (C, D) TEM images of nHAP, 5% Li‐nHAP, and 10% Li‐nHAP. (E, F) Length and diameter distributions. (G) TOF‐SIMS mapping of lithium signals. (H) ICP‐OES analysis of the time‐dependent Li/(Li + Ca) ratio in the release medium over 28 days. (I) FTIR spectra. (J) XRD patterns. (K) BET analysis. (L) pH‐dependent dissolution profiles of nHAP, 5%Li‐nHAP, and 10%Li‐nHAP under acidic to near‐physiological conditions.

Although lithium ions and biomimetic hydroxyapatite nanomaterials have been widely used for their therapeutic benefits, excessive lithium exposure may pose potential toxicity [53]. Therefore, it is crucial to determine the optimal lithium doping ratio and material concentration to balance biosafety and therapeutic efficacy. RAW 264.7 cells were treated with nHAP, 5%Li‐nHAP, and 10%Li‐nHAP at concentrations ranging from 0 to 8 µg/mL (0, 0.5, 1, 2, 4, and 8 µg/mL), and cell viability was evaluated by Cell Counting Kit‐8 (CCK‐8) assay. During the 5 days culture period, low to moderate concentrations, particularly 2 µg/mL, maintained RAW 264.7 cell viability comparable to the control group, whereas higher concentrations showed a mild inhibitory effect at later time points (Figure S3A–C). Based on these results, 2 µg/mL was selected for subsequent experiments on BMDMs. CCK‐8 assays revealed no significant difference in cell viability among all groups (Figure S3D). Moreover, all three materials exhibited low hemolysis rates at 2 µg/mL (Figure S3E,F). Considering the cytocompatibility and hemocompatibility results, 2 µg/mL was selected as the working concentration for in vitro experiments.

### 2.3 Li‐nHAP Rejuvenates Macrophages and Attenuates the Senescence‐Associated Secretory Phenotype (SASP)

Recent evidence highlights endogenous lithium as a vital regulator of myeloid immune homeostasis during aging, whose deficiency triggers pro‐inflammatory microglial activation and accelerates senescent pathologies [54]. To verify the anti‐senescence efficacy of Li‐nHAP, we performed transcriptomic analysis on Sn‐BMDMs treated with nHAP, Li‐nHAP or left untreated. Compared with Sn‐BMDMs, senescence‐related terms including cellular senescence, cell cycle, and the p53 signaling pathway were significantly enriched after Li‐nHAP treatment (Figure 3A). Similar enrichment was observed when comparing Li‐nHAP‐treated Sn‐BMDMs with nHAP‐treated counterparts (Figure S4A), confirming that the anti‐senescence efficacy is primarily attributed to Li^+^ rather than nHAP backbone. To extend our analysis, we interrogated the Aging Atlas database for curated senescence‐associated gene sets. The expression patterns of these genes in Li‐nHAP‐treated Sn‐BMDMs further support the anti‐senescence efficacy of Li‐nHAP (Figure S4B). GSEA further demonstrated that the senescent phenotype was markedly suppressed by Li‐nHAP, accompanied by enhanced activity of DNA replication, mismatch repair, PPAR signaling pathway [3], and cell cycle pathway (Figure S4C‐F).

**FIGURE 3:**
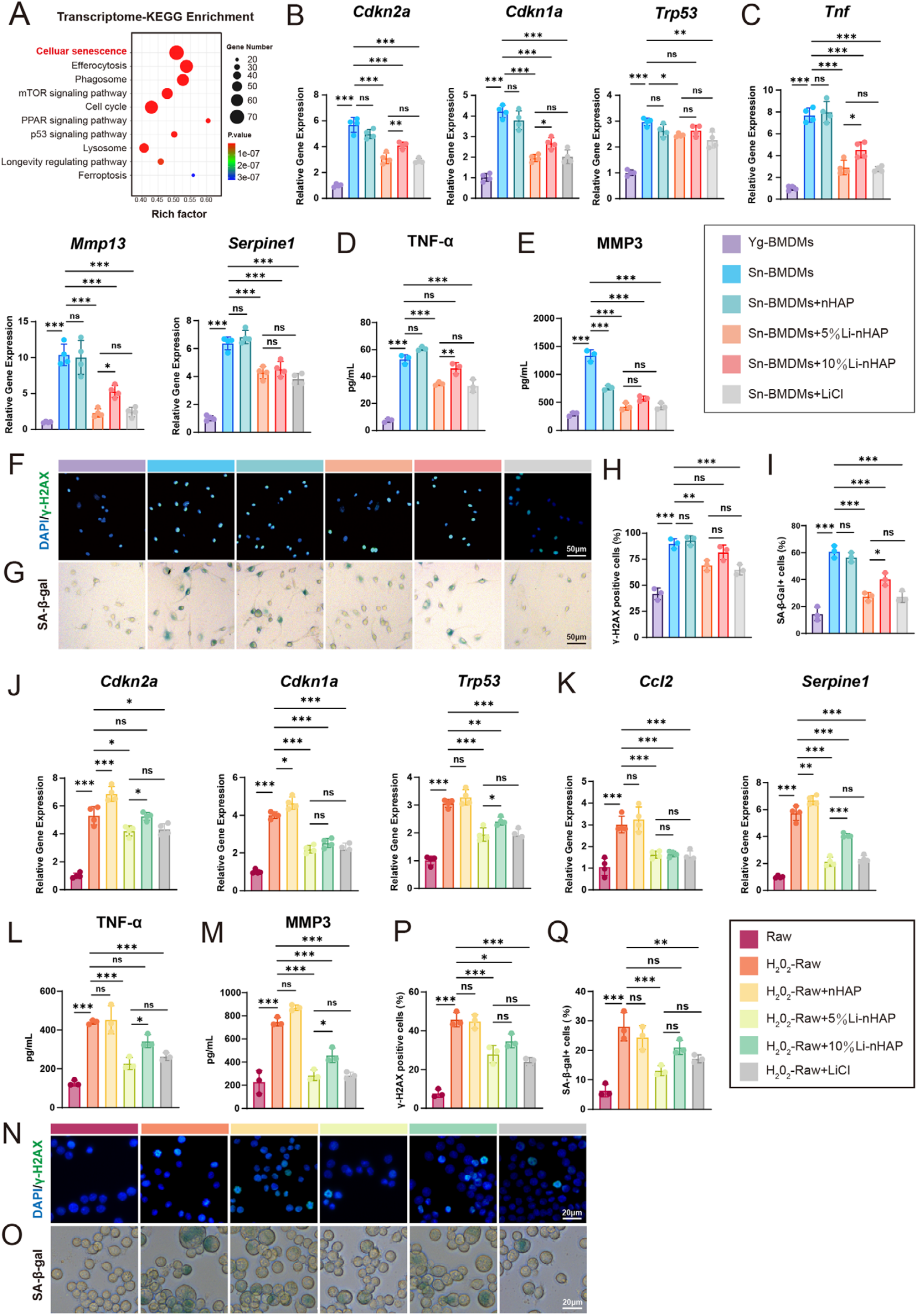
Li‐nHAP rejuvenates macrophages and attenuates the senescence‐associated secretory phenotype (SASP). (A) KEGG enrichment analysis comparing 5%Li‐nHAP‐treated Sn‐BMDMs with untreated Sn‐BMDMs. (B, C) Relative mRNA expression of senescence‐associated markers (B) and SASP factors (C) in BMDMs with different treatments. n = 4. (D, E) ELISA assessment of TNF‐α (D) and MMP3 (E) secretion in BMDMs culture supernatants. n = 3. (F, H) Representative γ‐H2AX immunofluorescence images (F) and quantitative analysis (H). n = 3. (G, I) Representative SA‐β‐gal staining (G) and quantitative analysis (I). n = 3. (J, K) Relative mRNA expression of senescence‐associated genes (J) and SASP‐related genes (K) in H_2_O_2_‐induced RAW 264.7 cells. n = 4. (L, M) ELISA assessment of TNF‐α (L) and MMP3 (M) secretion in culture supernatants. n = 3. (N, P) Representative γ‐H2AX immunofluorescence images (N) and quantitative analysis of γ‐H2AX‐positive cells (P). n = 3. (O, Q) Representative SA‐β‐gal staining (O) and quantitative analysis of SA‐β‐gal‐positive cells (Q). n = 3. Data are presented as mean ± SD. Statistical significance was determined by one‐way ANOVA followed by Tukey's post hoc test. ns, not significant; *p < 0.05, **p < 0.01, and ***p < 0.001.

To verify this, Sn‐BMDMs were treated with nHAP, 5%Li‐nHAP, or 10%Li‐nHAP and a series of senescence‐related markers were evaluated. Reverse transcription‐quantitative PCR (RT‐qPCR) analysis showed that both 5% and 10% Li‐nHAP significantly downregulated the senescence markers *Cdkn2a, Cdkn1a* and *Trp53* compared with Sn‐BMDMs group (Figure 3B). Consistently, the expression of SASP factors, including *Tnf*, *Mmp13* and *Serpine1*, was markedly reduced (Figure 3C). Notably, 5%Li‐nHAP elicited a more pronounced inhibitory effect than 10%Li‐nHAP, demonstrating its superior efficacy. Enzyme‐linked immunosorbent assay (ELISA) further confirmed that the secretion levels of MMP3 and TNF‐α were most notably decreased in 5%Li‐nHAP group (Figure 3D,E). Phosphorylated histone H2AX (γ‐H2AX) is a canonical marker of DNA oxidative damage associated with cellular senescence. Immunofluorescence staining revealed extensive γ‐H2AX foci accumulation in Sn‐BMDMs, which was markedly reduced upon 5%Li‐nHAP treatment (Figure 3F,H). Senescence‐associated β‐galactosidase (SA‐β‐gal) staining yielded consistent results (Figure 3G,I). To further validate these findings in an oxidative stress‐induced senescence model, RAW 264.7 cells were subsequently exposed to H_2_O_2_ [55]. H_2_O_2_ exposure markedly aggravated oxidative DNA damage and cellular senescence, which were significantly reversed by both 5% and 10% Li‐nHAP, as evidenced by reduced senescence‐associated gene expression (*Cdkn2a, Cdkn1a*, *Trp53, Ccl2* and *Serpine1*), decreased SASP secretion (MMP3 and TNF‐α), as well as diminished γ‐H2AX foci accumulation and SA‐β‐gal staining. Among them, 5%Li‐nHAP consistently exhibited the most pronounced protective effect (Figure 3J–Q). Collectively, these findings verify that 5%Li‐nHAP exerts superior protection against both replicative and oxidative stress‐induced macrophage senescence, outperforming nHAP and 10%Li‐nHAP. To dissect the specific contribution of Li^+^ from that of the nHAP nanowire backbone, an additional LiCl control group was included. Given that the 5% Li‐nHAP group demonstrated optimal performance and its Li^+^ release profile plateaued after approximately 21 days, the LiCl solution was formulated to match the cumulative Li^+^ release concentration of the 5% Li‐nHAP group at this time point. In Sn‐BMDMs, LiCl effectively mimicked the effects of 5% Li‐nHAP, as evidenced by the downregulation of senescence‐associated genes (*Cdkn2a*, *Cdkn1a*, and *Trp53*) and SASP factors (*Tnf*, *Mmp13*, and *Serpine1*), alongside reduced secretion of TNF‐α and MMP3, diminished γ‐H2AX accumulation, and decreased SA‐β‐gal activity (Figure 3B–I). These findings were further corroborated in H_2_O_2_‐induced senescent RAW 264.7 cells (Figure 3J–Q). In contrast, pristine nHAP exhibited only minimal effects across all assays. These findings indicate that the anti‐senescent activity of Li‐nHAP is primarily attributed to the bioactivity of released Li^+^, rather than to the nHAP backbone alone. On the basis of these results, the 5% doping ratio was therefore selected for all subsequent experiments and is referred to as Li‐nHAP hereafter.

To further characterize the immunomodulatory effects of Li‐nHAP, macrophage polarization was evaluated by RT‐qPCR analysis of representative polarization‐associated genes. LPS/IFN‐γ stimulation markedly increased the expression of M1 associated genes *Nos2*, *Tnf*, and *Il1b*. nHAP treatment did not significantly alter their expression, whereas Li‐nHAP markedly suppressed all three markers (Figure S5A–C). Conversely, under IL‐4 stimulation, Li‐nHAP further enhanced the expression of the M2 associated genes *Arg1* and *Il10* compared with IL‐4 alone or nHAP treatment (Figure S5D,E). These findings indicate that Li‐nHAP attenuates M1 polarization while favoring M2 polarization, further supporting its immunomodulatory capacity.

### 2.4 Li‐nHAP Alleviates Macrophage Senescence Through Ferroptosis Inhibition

Iron dyshomeostasis is increasingly recognized as a driver of cellular senescence, contributing to “ferro‐aging” [11]. Intriguingly, recent studies have demonstrated that lithium treatment possesses the capacity to counteract ferroptosis by reducing Fe^2^^+^ accumulation and limiting lipid peroxidation [49]. These findings prompted us to investigate whether Li‐nHAP alleviated macrophage senescence through ferroptosis modulation. To test this hypothesis, we further interrogated our multi‐omics data. Ingenuity Pathway Analysis (IPA) of transcriptomic and proteomic data revealed that the ferroptosis pathway was markedly activated in Sn‐BMDMs, whereas this activation was significantly attenuated upon Li‐nHAP treatment (Figure 4A). Combined transcriptomic and metabolomic analyses further corroborated these findings (Figure S4G). Proteomic profiling confirmed that Li‐nHAP upregulated the expression of anti‐ferroptotic proteins and downregulated the expression of pro‐ferroptotic proteins (Figure 4B). Taken together, these multi‐omics insights validated our hypothesis that Li‐nHAP alleviates macrophage senescence through modulation of ferroptosis.

**FIGURE 4:**
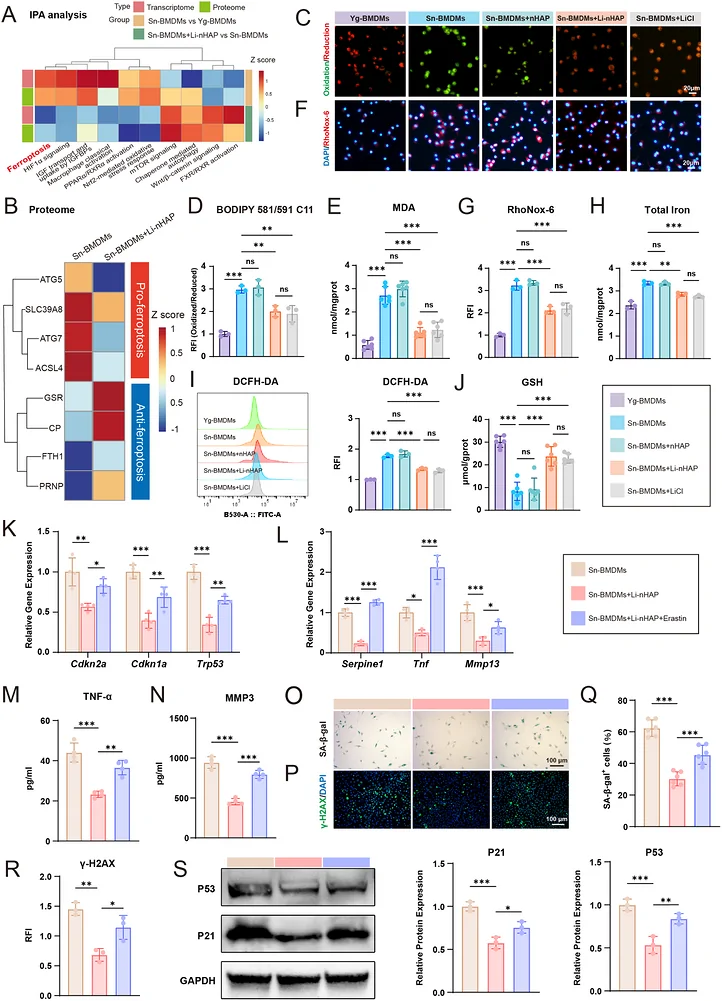
Li‐nHAP suppresses excessive ferroptosis to alleviate macrophage senescence. (A) IPA‐based pathway activation analysis of transcriptomic (n = 6) and proteomic data (n = 4). (B) Heatmap showing the mean abundances of selected pro‐ferroptosis and anti‐ferroptosis proteins in Sn‐BMDMs with or without Li‐nHAP treatment. n = 4. (C, D) BODIPY 581/591 C11 staining for lipid peroxidation (C) and the quantification (D). n = 3. (E) Intracellular MDA levels in BMDMs under different treatments. n = 6. (F, G) RhoNox‐6 probe for cytoplasmic Fe^2^^+^ accumulation (F) and the quantification (G). n = 3. (H) Quantification of total intracellular iron content. n = 3. (I) DCFH‐DA probe for intracellular ROS levels and the quantification. n = 3. (J) Intracellular GSH levels in BMDMs. n = 6. (K, L) Relative mRNA expression of senescence‐associated markers (K) and SASP factors (L). n = 3. (M, N) ELISA assessment of TNF‐α (M) and MMP3 (N). n = 4. (O‐R) SA‐β‐gal staining (O) (n = 6), γ‐H2AX immunofluorescence (P) (n = 4), and quantitative analysis (Q, R). (S) Western blotting analysis of P21 and P53 protein, and quantitative analysis. n = 3. Data are presented as mean ± SD. Statistical significance was determined by one‐way ANOVA followed by Tukey's post hoc test. ns, not significant; *p < 0.05, **p < 0.01, and ***p < 0.001.

The hallmarks of ferroptosis—characterized by iron‐catalyzed reactive oxygen species (ROS) generation and subsequent lipid peroxidation of polyunsaturated fatty acids [56]—were systematically evaluated in BMDMs with different treatments. BODIPY 581/591 C11 staining revealed conspicuous lipid peroxidation in Sn‐BMDMs, which was effectively reduced by Li‐nHAP (Figure 4C,D). Consistently, malondialdehyde (MDA) content, a terminal product of lipid peroxidation, was elevated in Sn‐BMDMs and significantly decreased upon Li‐nHAP intervention (Figure 4E). To elucidate the upstream drivers provoking this lipid peroxidation, intracellular ferrous ions (Fe^2+^) were monitored since Fe^2+^ serves as a key substrate of the Fenton reaction to generate highly toxic hydroxyl radicals by reacting with H_2_O_2_ [56]. The RhoNox‐6 probe revealed excessive Fe^2^^+^ accumulation in Sn‐BMDMs, which was markedly alleviated by Li‐nHAP (Figure 4F,G). Moreover, Sn‐BMDMs exhibited a marked increase in total iron content compared with Yg‐BMDMs. Li‐nHAP significantly reduced this elevation, demonstrating that Li‐nHAP ameliorates age‐related iron dyshomeostasis (Figure 4H). DCFH‐DA staining further confirmed that the massive intracellular ROS in Sn‐BMDMs was profoundly attenuated by Li‐nHAP (Figure 4I). Glutathione (GSH) is a major intracellular antioxidant and an essential component of the cellular defense against ferroptosis [57]. GSH levels were markedly reduced in Sn‐BMDMs but were partially restored by Li‐nHAP treatment (Figure 4J). Notably, LiCl treatment produced effects comparable to those of Li‐nHAP, while pristine nHAP treatment exerted no obvious effects on the ferroptotic phenotype of Sn‐BMDMs across all assays (Figure 4C‐J). These findings further support released Li^+^ as the major contributor to the anti‐ferroptotic effect of Li‐nHAP.

The interplay between cellular senescence and iron dysregulation highlights ferroptosis as a therapeutic target for senescence reversal [11, 58]. To verify whether Li‐nHAP exerted its anti‐senescence effect via ferroptosis inhibition, we used the well‐established ferroptosis inducer Erastin in rescue experiments. RT‐qPCR analysis revealed that Erastin effectively abolished the Li‐nHAP‐mediated downregulation of senescence markers and SASP‐related genes (Figure 4K,L). ELISA assay further confirmed that the Li‐nHAP induced reduction of TNF‐α and MMP3 secretion was significantly blocked by Erastin (Figure 4M,N). Consistently, the proportions of SA‐β‐gal‐positive and γ‐H2AX‐positive cells were markedly higher in the Li‐nHAP+Erastin group than in the Li‐nHAP group (Figure 4O‐R). Finally, western blotting analysis confirmed that Erastin partially compromised the ability of Li‐nHAP to downregulate P21 and P53 protein levels (Figure 4S). Collectively, these rescue experiments demonstrated that Li‐nHAP inhibits macrophage senescence primarily through suppression of the ferroptosis pathway.

### 2.5 Li‐nHAP Mitigates Macrophage Ferroptosis by Restoring Lysosomal Homeostasis

Intracellular iron homeostasis is regulated by multiple organelles, which control iron absorption, storage, utilization, and efflux through a complex regulatory network involving the mitochondria and lysosomes [59]. Mitophagy modulates mitochondrial iron content by regulating the synthesis of iron–sulfur clusters and heme production, thereby affecting overall cellular iron homeostasis. Conversely, ferritinophagy releases free iron by degrading the iron storage protein ferritin, which elevates the intracellular labile iron pool and subsequently promotes ferroptosis [60]. Moreover, reduced mitophagy may lead to the accumulation of damaged mitochondria and increased mitochondrial reactive oxygen species (mtROS), further facilitating ferroptotic cell death [61]. To determine whether Li‐nHAP regulates ferroptosis through mitochondrial pathways, mitophagy markers (*Sqstm1, Map1lc3b, Becn1*, and *Pink1*) and mitochondrial iron/ROS‐related genes (*Slc25a28* and *Dhodh*) were examined. These results did not reveal transcriptional changes following Li‐nHAP treatment (Figure S6A–F).

To identify the actual subcellular target, the multi‐omics dataset was re‐interrogated. IPA analysis revealed that while mitochondrial networks remained largely unperturbed, prominent transcriptomic and proteomic alterations uniquely converged on the lysosomal compartment comparing Sn‐BMDMs with Yg‐BMDMs (Figure S7). Previous studies have established lysosomal membrane peroxidation and subsequent labile iron leakage as upstream triggers of ferroptosis [18, 62]. Lithium inhibits glycogen synthase kinase‐3β (GSK‐3β) [35], which in turn promotes the nuclear translocation of Transcription Factor EB (TFEB)—a master regulator of lysosomal biogenesis [36]. Hence, the investigation was redirected toward this lysosome‐dependent ferroptosis axis. AUCell analysis of the GSE280914 dataset further revealed that the lysosomal functional score was markedly reduced in macrophages within aged bone defect (Figure 5A), accompanied by decreased expression of lysosome‐related ATPases (Figure 5B). GO enrichment analysis of our proteomics data revealed that lysosome‐related pathways—including acidification, membrane permeability, and protein catabolism—were significantly enriched after Li‐nHAP treatment (Figure 5C). These findings prompted a systematic evaluation of lysosomal homeostasis in Sn‐BMDMs.

**FIGURE 5:**
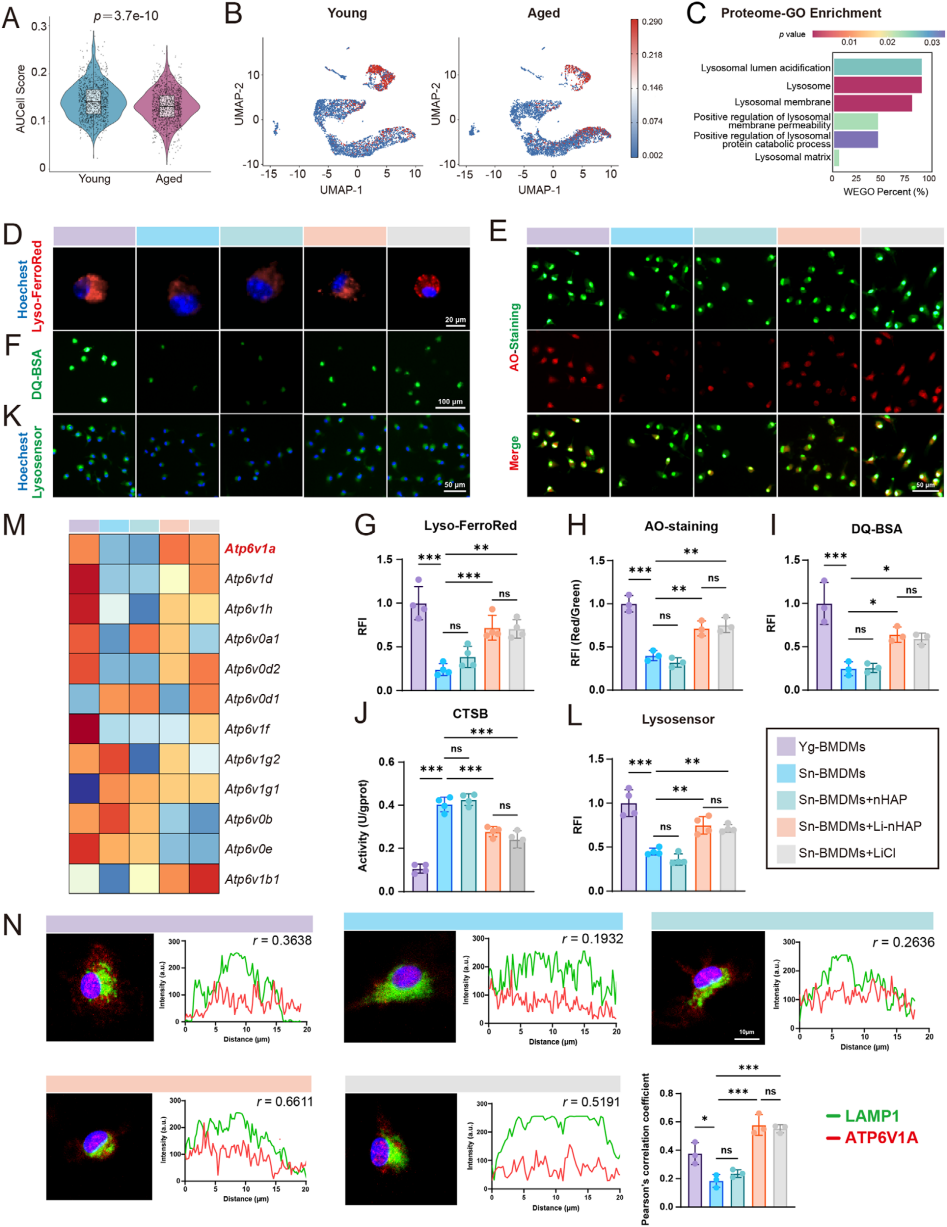
Li‐nHAP restores lysosomal homeostasis in Sn‐BMDMs. (A) AUCell analysis of lysosomal function in macrophages from the GSE280914 scRNA‐seq dataset. (B) Expression of lysosomal ATPase‐related genes in macrophages from young and aged mice in the GSE280914 scRNA‐seq dataset. (C) GO enrichment analysis of lysosome‐related pathways in Sn‐BMDMs following Li‐nHAP treatment. (D, G) Representative Lyso‐FerroRed fluorescence images (D) and quantification of the lysosomal labile Fe^2^^+^ signal (G). n = 4. (E, H) Representative Acridine Orange (AO) staining images (E) and quantitative analysis of lysosomal membrane integrity (H). n = 3. (F, I) Representative DQ‐BSA fluorescence images (F) and quantitative analysis of lysosomal proteolytic activity (I). n = 3. (J) Cathepsin B (CTSB) activity in BMDMs. n = 4. (K, L) Representative LysoSensor fluorescence images (K) and quantitative analysis of lysosomal acidity (L). n = 4. (M) RT‐qPCR heatmap showing the expression of V‐ATPase subunits in BMDMs. n = 4. The heatmap shows mean expression levels. (N) Representative immunofluorescence images, line‐scan fluorescence intensity profiles, and quantitative analysis of the colocalization between ATP6V1A and the lysosomal marker LAMP1. n = 3. Data are presented as mean ± SD. AUCell scores were compared using a two‐sided Wilcoxon rank‐sum test. Other statistical comparisons were performed using one‐way ANOVA followed by Tukey's post hoc test. ns, not significant; *p < 0.05, **p < 0.01, and ***p < 0.001.

Lysosomal labile Fe^2^^+^ pool was first examined using the Lyso‐FerroRed probe. Lysosomal iron content was remarkably depleted in Sn‐BMDMs, whereas Li‐nHAP treatment effectively restored this pool (Figure 5D,G). Intriguingly, this lysosomal iron deficit corresponded with the increased cytoplasmic iron levels, suggesting lysosomal leakage as the source of excess cytoplasmic iron in Sn‐BMDMs (Figure 4F,G). Lysosomal membrane permeability is another key hallmark of lysosomal dysfunction, which was next assessed using Acridine Orange (AO) staining. The results revealed increased lysosomal membrane permeability in Sn‐BMDMs, which was partially restored by Li‐nHAP treatment (Figure 5E,H). Since the leakage of lysosomal iron and increased lysosomal membrane permeabilization may imply the disruption of lysosomal homeostasis, the metabolic function of lysosomes was further examined. As expected, DQ‐BSA staining indicated that the decline in lysosomal hydrolytic activity in Sn‐BMDMs was reversed by Li‐nHAP (Figure 5F,I). The activity of cathepsin B (CTSB) was also alleviated by Li‐nHAP, which was abnormally elevated in senescent macrophages [63] (Figure 5J). Given that age‐related decline in lysosomal acidification was reported to destabilize lysosomal membrane integrity [9], lysosomal acidity was evaluated using the Lysosensor probe. Lysosomal acidity was markedly reduced in Sn‐BMDMs, and this acidification defect was mitigated by Li‐nHAP treatment (Figure 5K, L). RT‐qPCR analysis revealed that the expression of multiple V‐ATPase subunits (*Atp6v1a*, *Atp6v1d*, *Atp6v1h*, *Atp6v0a1, Atp6v0d2*) was significantly decreased in Sn‐BMDMs, while Li‐nHAP intervention markedly rescued the expression of *Atp6v1a* (Figure 5M). ATP6V1A is an essential V1 subunit of V‐ATPase and plays a critical role in maintaining lysosomal acidification and function [64, 65]. Simultaneously, immunofluorescence staining further confirmed that the colocalization of ATP6V1A with the lysosomal marker LAMP1 declined during senescence and was significantly restored after Li‐nHAP treatment (Figure 5N). Collectively, these findings demonstrated that senescence induces lysosomal dysfunction, while Li‐nHAP effectively restores lysosomal homeostasis across multiple dimensions—maintaining iron storage, preserving membrane integrity, enhancing hydrolytic activity, and restoring acidity. Importantly, LiCl solution largely recapitulated the lysosomal protective effects of Li‐nHAP. LiCl restored the lysosomal labile Fe^2^^+^ pool, improved lysosomal membrane integrity and proteolytic activity, partially normalized CTSB activity, and restored lysosomal acidification to levels comparable to those achieved by Li‐nHAP (Figure 5D–L). Consistent effects were also observed for V‐ATPase‐related expression and ATP6V1A–LAMP1 colocalization (Figure 5M,N). In contrast, pristine nHAP exerted limited effects on these lysosomal abnormalities. These findings further implicate released Li^+^ as the key bioactive factor underlying the lysosomal restoration induced by Li‐nHAP.

To test whether the anti‐ferroptotic effect of Li‐nHAP causally depended on lysosomal functional restoration, we performed rescue experiments with bafilomycin A1 (BafA1) (a lysosomal acidification inhibitor) and L‐leucyl‐L‐leucine methyl ester (LLOME) (a lysosomal membrane‐disrupting agent) [18]. BafA1 treatment significantly impeded the Li‐nHAP‐mediated suppression of ferroptosis, as evidenced by increased cytoplasmic Fe^2^^+^ accumulation (Figure 6A,B,F), elevated lipid peroxidation (Figure 6C,G), enhanced ROS production (Figure 6D,H), elevated MDA content (Figure 6I), decreased GPX4 expression (Figure 6E), and reduced GSH content (Figure 6J). Consistently, parallel experiments with LLOME yielded identical trends across all ferroptotic parameters (Figure 6K–T). These rescue experiments established that the anti‐ferroptotic function of Li‐nHAP depends on intact lysosomal homeostasis.

**FIGURE 6:**
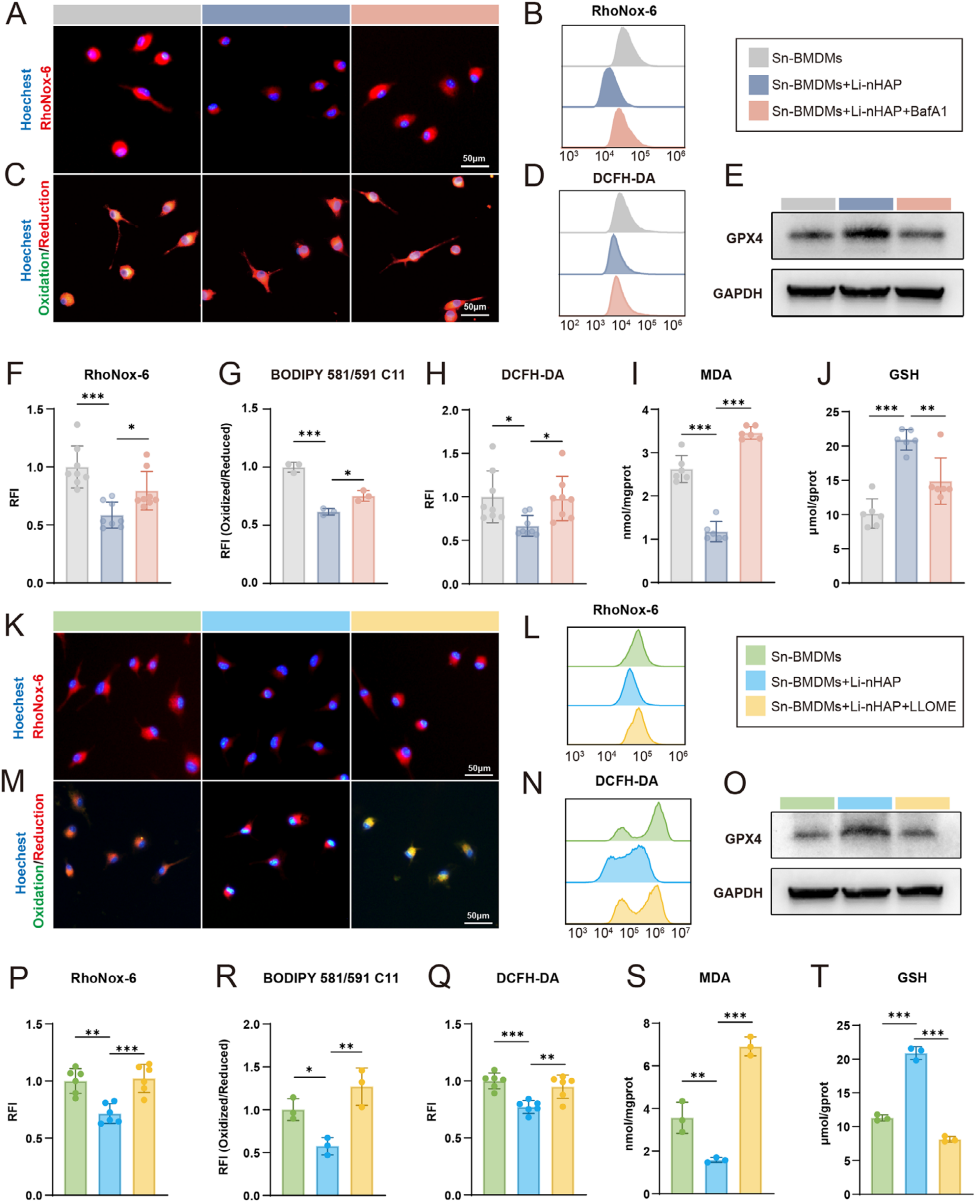
Blockade of lysosomal function abolishes the anti‐ferroptotic effect of Li‐nHAP in Sn‐BMDMs. (A–J) Evaluations of ferroptosis‐related parameters in Sn‐BMDMs co‐treated with Li‐nHAP and BafA1. (A, B, F) Cytoplasmic iron was assessed by RhoNox‐6 staining (A), flow cytometry (B), and the quantification (F). n = 8. (C, G) BODIPY 581/591 C11 staining for lipid peroxidation (C) and the quantification (G). n = 3. (D, H) DCFH‐DA probe for intracellular ROS levels (D) and the quantification (H). n = 8. (E) Western blotting of GPX4 protein expression. (I, J) Intracellular MDA (I) and GSH (J) levels. n = 6. (K–T) Parallel evaluations in Sn‐BMDMs following co‐treatment with Li‐nHAP and LLOME. (K, L, P) Cytoplasmic iron was assessed by RhoNox‐6 staining. n = 6. (M, R) BODIPY 581/591 C11 staining for lipid peroxidation. n = 3. (N, Q) DCFH‐DA probe for intracellular ROS levels. n = 6. (O) Western blotting of GPX4 protein expression. (S, T) Intracellular MDA (S) and GSH levels (T). n = 3. Data are presented as mean ± SD. Statistical significance was determined by one‐way ANOVA followed by Tukey's post hoc test. *p < 0.05, **p < 0.01, and ***p < 0.001.

### 2.6 Li‐nHAP Promotes Osteogenic Differentiation of Senescent BMSCs Through Macrophage‐Mediated Paracrine Signaling

Previous studies have demonstrated that senescent macrophages secrete excessive levels of osteoinhibitory factors, thereby impairing in vivo bone defect healing [5]. In addition to traditional SASP factors, ferroptotic cells release damage‐associated molecular patterns (DAMPs) and lipid peroxides into the microenvironment, which can trigger secondary senescence in neighboring stem cells and impair osteogenesis [13, 66]. Given the capability of Li‐nHAP to alleviate macrophage senescence, we next investigated whether the paracrine factors from Li‐nHAP‐rejuvenated macrophages could promote osteogenesis. To this end, senescent BMSCs were cultured in CM derived from BMDMs subjected to different treatments, and osteogenic differentiation was evaluated after induction (Figure 7A). Immunofluorescence staining revealed that compared with Yg‐BMDMs CM, Sn‐BMDMs CM significantly suppressed osteopontin (OPN) expression in senescent BMSCs. Strikingly, CM from Sn‐BMDMs treated with Li‐nHAP (Sn‐BMDMs+Li‐nHAP CM) effectively reversed this suppression, while the CM from Sn‐BMDMs treated with nHAP (Sn‐BMDMs+nHAP CM) showed only a weak effect (Figure 7B,F). Furthermore, alkaline phosphatase (ALP) staining on day 10 and Alizarin Red S (ARS) staining on day 21 were performed to evaluate early osteogenic differentiation and late‐stage mineralization respectively. As expected, BMSCs cultured in Sn‐BMDMs+Li‐nHAP CM showed increased ALP staining (Figure 7C,G) and the formation of mineralized nodules (Figure 7D,H) compared with Sn‐BMDMs CM. RT‐qPCR analysis further corroborated these findings, demonstrating that Sn‐BMDMs+Li‐nHAP CM treatment significantly increased the expression of osteogenic genes (*Runx2*, *Alpl*, *Spp1*, and *Col1a1*) in senescent BMSCs (Figure 7E). Consistently, LiCl treatment largely recapitulated the effects of Li‐nHAP. Collectively, these findings indicate that CM from Li‐nHAP‐treated Sn‐BMDMs provides a more favorable paracrine environment for the osteogenic differentiation of senescent BMSCs.

**FIGURE 7:**
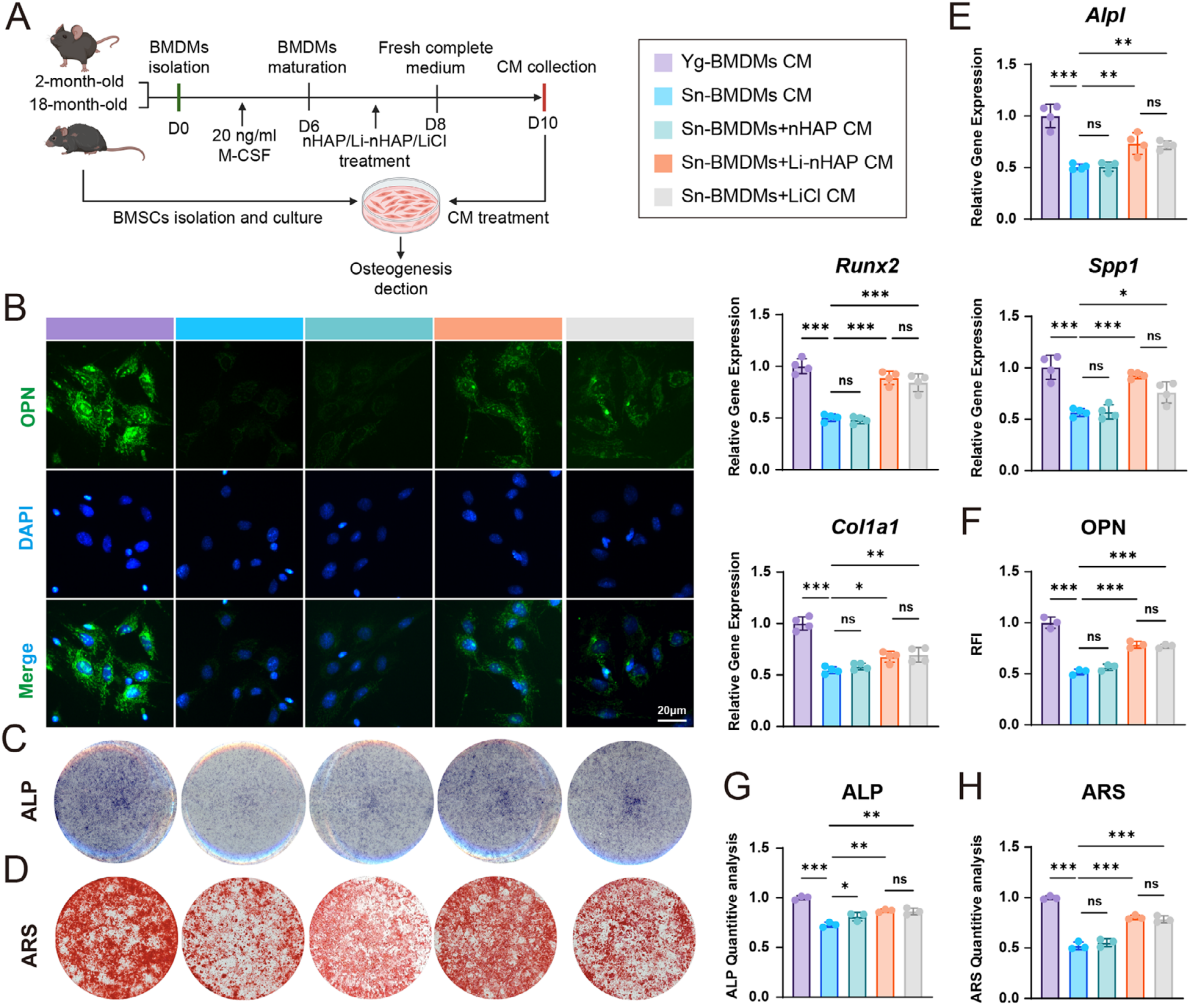
Li‐nHAP promotes osteogenic differentiation of senescent BMSCs through macrophage‐mediated paracrine signaling. (A) Schematic illustration of the experimental design. (B, F) Immunofluorescence assay for OPN expression (B) and corresponding quantitative analysis (F). n = 3. (C, D, G, H) ALP and ARS staining in senescent BMSCs (C, D), with corresponding quantitative analysis (G, H). n = 3. (E) Relative mRNA expression of osteogenic genes (*Runx2*, *Alpl*, *Spp1* and *Col1a1*) in senescent BMSCs. n = 4. Data are presented as mean ± SD. Statistical significance was determined by one‐way ANOVA followed by Tukey's post hoc test. ns, not significant; *p < 0.05, **p < 0.01, and ***p < 0.001.

### 2.7 Li‐nHAP+GelMA Promotes Repair of Critical‐Size Mandibular Defects in Aged Mice

For clinical translation, the systemic use of lithium is hampered by its narrow therapeutic window and potential off‐target toxicity, despite its well‐established psychiatric efficacy and benefits at low doses [32, 67]. To evaluate the in vivo performance of the nanowires, nHAP or Li‐nHAP was encapsulated within a gelatin methacryloyl (GelMA) hydrogel to construct a localized and sustained delivery system. In this integrated platform, nHAP not only serves as a reservoir for sustained Li^+^ release but also provides a bone‐mimetic and osteoconductive mineral phase resembling the inorganic component of native bone. GelMA provides a hydrated extracellular matrix‐like microenvironment that supports cellular infiltration and prevents burst release, making it an ideal carrier to enhance local retention of incorporated Li‐nHAP [68, 69]. Thus, the in vivo regenerative efficacy of Li‐nHAP+GelMA is likely attributable to the convergence of Li^+^‐mediated bioactivity and the favorable delivery properties of the material platform. SEM analysis confirmed that loading GelMA with nHAP or Li‐nHAP preserved its loose and porous architecture (Figure S8A), which is essential for facilitating the infiltration of immune cells and regenerating tissues. Subsequently, the critical‐size mandibular defect model was established in aged mice [70], and the defects were randomly filled with GelMA, nHAP+GelMA or Li‐nHAP+GelMA, with Blank serving as the control. Macroscopic observation confirmed the successful creation of the defects and successful implantation of the hydrogels (Figure 8A). The hematological parameters (WBC, RBC, PLT), liver function markers (ALT, AST), renal function indicators (BUN, CR), and plasma LDH and CK levels showed no significant differences among all the groups (Figure S8B‐J). Furthermore, hematoxylin and eosin (H&E) staining of major organs (heart, liver, spleen, lung, and kidney) revealed no notable histopathological changes across all groups (Figure S8K). These results collectively confirm the favorable in vivo biosafety profile of Li‐nHAP+GelMA.

**FIGURE 8:**
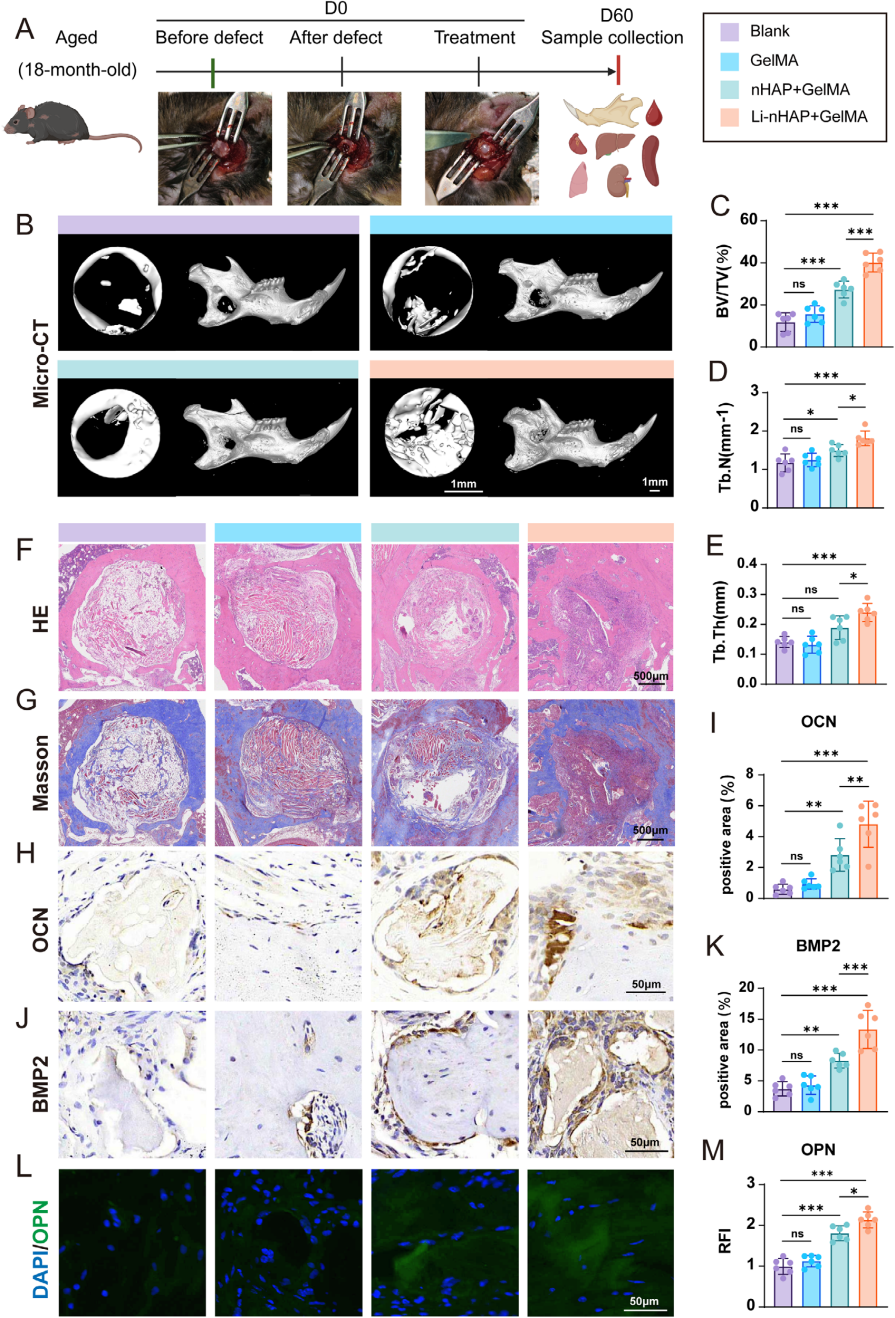
Li‐nHAP+GelMA promotes critical‐size mandibular defect repair in aged mice. (A) Schematic illustration of the experimental design and macroscopic photographs of the mandibular defect model and material implantation. (B) Representative 3D reconstructed Micro‐CT images. (C–E) Quantitative analysis of bone volume/total volume (BV/TV) (C), trabecular number (Tb.N) (D), and trabecular thickness (Tb.Th) (E). n = 6. (F, G) Representative histological evaluation of new bone formation revealed by H&E staining (F) and Masson's trichrome staining (G). (H–K) Representative immunohistochemical staining and quantification of OCN (H, I) and BMP2 (J, K). n = 6. (L, M) Representative immunofluorescence staining of OPN (L) and quantification (M). n = 6. Data are presented as mean ± SD. Statistical significance was determined by one‐way ANOVA followed by Tukey's post hoc test. ns, not significant; *p < 0.05, **p < 0.01, and ***p < 0.001.

Micro‐CT scanning and 3D reconstruction were performed at 8 weeks after surgery. The results showed that the defects in Blank and GelMA groups were poorly healed, with only sparse bone formation at the defect margins, indicating severely impaired regenerative capacity of aged bone and the limited osteoinductivity of GelMA. The nHAP+GelMA group exhibited moderate bone repair, whereas the Li‐nHAP+GelMA group showed more pronounced bone defect regeneration (Figure 8B). Furthermore, the Li‐nHAP+GelMA group exhibited significantly higher bone volume/total volume (BV/TV), trabecular number (Tb.N), and trabecular thickness (Tb.Th) compared with the other three groups (Figure 8C‐E). Histological evaluations, including H&E and Masson's trichrome staining supported the above findings. Compared with the Blank and GelMA groups, which were predominantly filled with fibrous connective tissue, the nHAP+GelMA group exhibited moderate new bone formation and matrix mineralization, whereas the Li‐nHAP+GelMA group displayed robust trabecular bone formation and mature bone matrix mineralization (Figure 8F‐G). Consistently, immunohistochemical analysis of osteogenic markers within the defect area revealed an increase in osteocalcin (OCN) and bone morphogenetic protein 2 (BMP2) expression from the Blank/GelMA groups to the nHAP+GelMA group, with the Li‐nHAP+GelMA group exhibiting the most pronounced upregulation (Figure 8H‐K). Immunofluorescence staining of OPN corroborated this trend (Figure 8L,M). Collectively, these findings demonstrated that Li‐nHAP+GelMA effectively rescues bone healing in the critical‐size mandibular defect in aged mice.

To further evaluate whether the regenerative benefit of Li‐nHAP+GelMA was maintained during the later stage of bone healing, mandibular defects were additionally assessed at 12 weeks post‐surgery. Micro‐CT reconstruction revealed persistent and extensive bone regeneration in the Li‐nHAP+GelMA group, whereas substantially less defect filling was observed in the Blank and GelMA groups (Figure S9A). Quantitative analysis further demonstrated that the Li‐nHAP+GelMA group exhibited significantly higher BV/TV, Tb.N, and Tb.Th, with superior values also observed compared with the nHAP+GelMA group (Figure S9B–D). Consistently, immunohistochemical analysis demonstrated robust and sustained osteogenic activity in the Li‐nHAP+GelMA group, as reflected by significantly increased OCN and BMP2 positive regions (Figure S9E–H). These findings further support the superior regenerative efficacy of Li‐nHAP+GelMA during the later stage of aged mandibular repair.

### 2.8 Li‐nHAP+GelMA Ameliorates Macrophage Ferroptosis and Senescence in Aged Mandibular Defects

Having established the superior osteogenic capacity of Li‐nHAP+GelMA, we next sought to determine whether this regenerative effect was associated with modulation of macrophage ferroptosis and senescence, as delineated in our findings in vivo. In the Blank, GelMA and nHAP+GelMA groups, a substantial fraction of F4/80^+^ macrophages within the defect region exhibited strong P21 and γ‐H2AX signals, indicating macrophage senescence and DNA damage in the aged regenerative microenvironment (Figure 9A,B). By contrast, Li‐nHAP+GelMA markedly reduced the overlap between F4/80 and these senescence‐associated markers, indicating the attenuation of macrophage senescence in vivo. To further determine whether Li‐nHAP+GelMA regulated macrophage ferroptosis in the aged defect microenvironment, we examined GPX4 expression and 4‐hydroxynonenal (4‐HNE) accumulation in F4/80^+^ macrophages. GPX4 signal in macrophage‐rich regions was relatively weak in the Blank, GelMA, and nHAP+GelMA groups but was restored after Li‐nHAP+GelMA treatment (Figure 9C). Conversely, 4‐HNE accumulation was prominent in the Blank, GelMA, and nHAP+GelMA groups, whereas Li‐nHAP+GelMA substantially reduced this lipid peroxidation signal (Figure 9D). Abnormal iron accumulation served as a hallmark of the senescent microenvironment [11]. Prussian blue staining further showed obvious iron deposition within the defect area in the Blank, GelMA, and nHAP+GelMA groups, which was decreased in the Li‐nHAP+GelMA group (Figure 9E). These in vivo findings align with our in vitro mechanistic data and indicate that Li‐nHAP+GelMA revitalizes the aged defect microenvironment by suppressing macrophage senescence and excessive ferroptosis.

**FIGURE 9:**
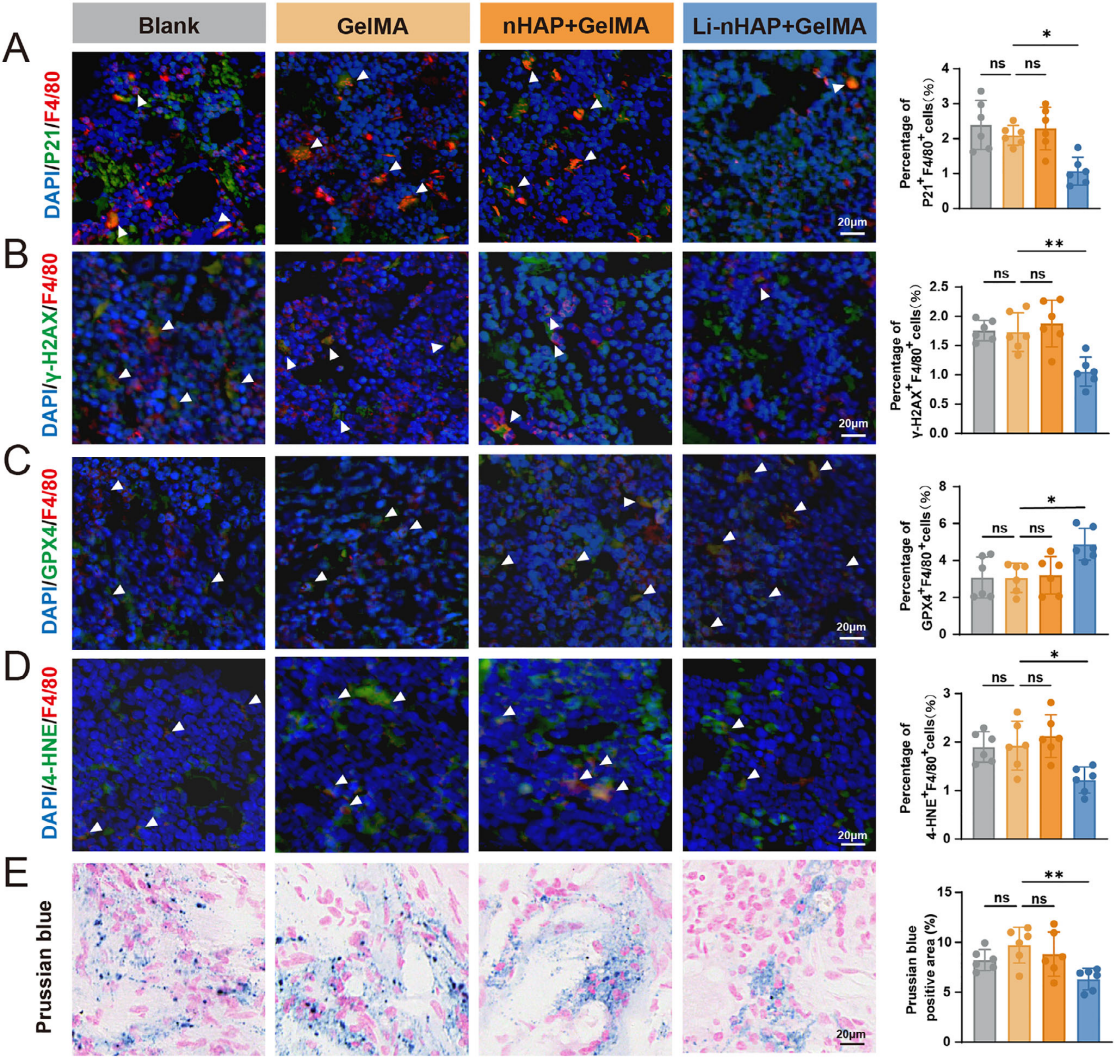
Li‐nHAP+GelMA revitalizes the aged microenvironment by inhibiting macrophage senescence and excessive ferroptosis in vivo. (A‐D) Dual‐labeling immunofluorescence of macrophages (F4/80^+^, red) co‐stained with senescence marker P21 (green) (A), DNA damage marker γ‐H2AX (green) (B), anti‐ferroptotic protein GPX4 (green) (C) and lipid peroxidation marker 4‐HNE (green) (D) and the quantification. Nuclei were counterstained with DAPI (blue). n = 6. (E) Representative Prussian blue staining for localized iron accumulation, and the quantification. n = 6. Data are presented as mean ± SD. Statistical significance was determined by one‐way ANOVA followed by Tukey's post hoc test. ns, not significant; *p < 0.05, **p < 0.01.

To determine whether the regenerative efficacy of Li‐nHAP+GelMA depends on macrophages, we depleted macrophages in vivo using clodronate liposomes. F4/80 immunofluorescence confirmed effective macrophage depletion within the mandibular defect region (Figure S10A). At 8 weeks, micro‐CT analysis revealed that macrophage depletion significantly reduced BV/TV, Tb.N, and Tb.Th compared with control liposomes. Notably, the osteogenic effect of Li‐nHAP+GelMA was markedly attenuated in macrophage‐depleted mice, suggesting that its regenerative efficacy is partially dependent on the presence of macrophages (Figure S10B–E). To further assess the contribution of ferroptosis in vivo, we pharmacologically activated ferroptosis using Erastin. Compared with the GelMA group, Erastin significantly impaired bone regeneration, as reflected by reductions in BV/TV, Tb.N, and Tb.Th at 8 weeks. Moreover, Li‐nHAP+GelMA failed to promote bone defect repair under Erastin treatment, indicating ferroptosis suppression as a key mechanism (Figure S11). Collectively, the macrophage depletion and ferroptosis activation experiments provide complementary in vivo evidence that the regenerative advantage of Li‐nHAP+GelMA is partially dependent on macrophages and is critically linked to the inhibition of excessive ferroptosis.

Despite these promising findings, several limitations should be acknowledged. First, although macrophage depletion and pharmacological activation of ferroptosis provided functional in vivo evidence for the roles of macrophages and ferroptosis in Li‐nHAP+GelMA mediated bone regeneration, these experiments do not directly establish macrophage‐specific genetic causality. In addition, the potential effects of locally delivered lithium on other components of the osteoimmune microenvironment, including T lymphocytes [71], endothelial cells [72], and osteoclasts [73], remain to be fully defined. Future studies using macrophage‐specific genetic manipulation of ferroptosis regulators will be useful for further defining the specific role of macrophage ferroptosis in this process. Second, to avoid the confounding effects of sexual dimorphism on immune aging and bone regeneration, we selected male mice for experiments. In the future, parallel studies in aged female animals are needed to evaluate sex‐related differences in Li‐nHAP efficacy [74, 75]. Finally, the local concentration of Li^+^ released within the bone defect microenvironment was not directly measured in vivo. Future studies should directly assess both the local and systemic levels of Li^+^ to better characterize the in vivo release profile of Li‐nHAP.

## 3 Conclusion

Macrophage senescence is a critical determinant of impaired bone regeneration in the aged skeleton. In this study, guided by multi‐omics evidence of lysosomal dysfunction and ferroptosis dysregulation in Sn‐BMDMs, we engineered lithium‐doped hydroxyapatite nanowires (Li‐nHAP) to counteract this phenotype. In vivo experiments demonstrated that Li‐nHAP effectively rejuvenates Sn‐BMDMs and promotes the osteogenic differentiation of aged BMSCs. Mechanistically, the sustained release of lithium ions from Li‐nHAP re‐establishes lysosomal homeostasis, including restoring acidity, preserving membrane integrity, maintaining iron storage, and enhancing hydrolytic activity, thereby blocking ferroptosis and alleviating macrophage senescence. In an aged mouse mandibular defect model, Li‐nHAP+GelMA significantly reduced iron overload, suppressed macrophage senescence and ferroptosis, and achieved superior in vivo bone regeneration. Here, we identify lysosomal iron dyshomeostasis as a ferroptosis‐linked feature of Sn‐BMDMs during aged bone repair and demonstrate that locally delivered Li‐nHAP reprograms this macrophage state to promote mandibular regeneration in aged mice.

## 4 Experimental Section

### 4.1 Preparation and Characterization of nHAP and Li‐Doped nHAP

Briefly, LiNO_3_, Ca(NO_3_)_2_·4H_2_O, and Na_2_SiO_3_·9H_2_O (Aladdin, China) were dissolved to prepare a homogeneous mixed solution, which was then subjected to hydrothermal treatment to obtain the precursor. The molar ratio of lithium to the total content of calcium and lithium was adjusted to 0%, 5%, and 10%. The resulting precursor was collected by suction filtration, rinsed sequentially with deionized water and absolute ethanol, and dried to yield precursor powder. Subsequently, the precursor was blended with sodium phosphate dodecahydrate solution and subjected to a second hydrothermal reaction to synthesize nHAP, 5%Li‐nHAP, and 10%Li‐nHAP. The final products were thoroughly washed and dried. For SEM characterization, samples were mounted on conductive adhesive tape and sputter‐coated with gold for 45 s using a SC7620 sputtering coater (Quorum, UK). Surface morphology was observed using a GeminiSEM 300 scanning electron microscope (ZEISS, Germany) at an accelerating voltage of 3 kV with an SE2 secondary electron detector. For TEM, samples were dispersed in ethanol, ultrasonicated, and dropped onto copper grids. After air drying, morphology was examined using a JEM‐F200 transmission electron microscope (JEOL, Japan) at 200 kV, equipped with a JED‐2300T energy dispersive spectrometer (JEOL, Japan). TOF‐SIMS analysis was performed on a PHI nanoTOF II instrument (ULVAC‐PHI, Japan) using a Bi_3_^+^ primary ion source (30 keV, 2 nA). The raster size was 100 µm × 100 µm, and spectra were acquired in high mass resolution mode over a mass range of 1–1850 u. Sputtering was conducted with an Ar^+^ ion gun (3 kV, 100 nA) over a 400 µm × 400 µm area. ICP‐OES analysis was carried out using Thermo Fisher iCAP PRO OES instrument (Thermo Fisher, USA). Standard solutions with gradient concentrations were prepared to generate a calibration curve, and sample solutions were then quantitatively analyzed. FTIR spectra were recorded on a Thermo Fisher Nicolet iS20 spectrometer (Thermo Fisher, USA). Samples were ground with dry KBr powder in an agate mortar and pressed into transparent thin sheets. Spectra were collected at a resolution of 4 cm^−^^1^, with 32 scans over a wavenumber range of 400–4000 cm^−^^1^, following background subtraction. XRD patterns were obtained using a Bruker D8 Advance diffractometer (Bruker, Germany) with Cu‐Kα radiation (40 kV, 40 mA). Samples were scanned from 10° to 80° at a rate of 5°/min. BET surface area analysis was performed on a Micromeritics ASAP 2460 instrument (Micromeritics, USA). Samples were degassed under vacuum at 120°C for 8 h, and nitrogen adsorption–desorption isotherms were measured at 77 K. The specific surface area was calculated using the BET model. For pH‐dependent solubility measurements, excess nHAP, 5% Li‐nHAP, and 10% Li‐nHAP were separately added to 70 mL aqueous solutions adjusted to designated pH values. The suspensions were then filtered, and the Ca^2^^+^ concentrations in the filtrates were quantified by cation‐exchange column separation to calculate the dissolved amount of each material.

### 4.2 Preparation of nHAP‐ or Li‐nHAP‐Incorporated GelMA Hydrogels

To construct the localized delivery system, GelMA (Engineering For Life, China) and the photoinitiator lithium phenyl‐2,4,6‐trimethylbenzoylphosphinate (LAP) were dissolved in PBS at final concentrations of 10% (w/v) and 0.25% (w/v), respectively, under continuous stirring at 40°C. Subsequently, nHAP or Li‐nHAP were homogeneously dispersed into the GelMA prepolymer solution. For in vivo application, the composite prepolymer solution was administered to the defect site and crosslinked via 405 nm blue light for 30 s to form a stable hydrogel network. For in vivo ferroptosis induction, Erastin (TargetMol, China) was locally delivered via encapsulation into the GelMA hydrogel system according to the manufacturer's instructions.

### 4.3 Ethics Approvals and Consent to Participate

All animal procedures, including cell isolation and animal experiments, were approved by the Ethics Committee of Shanghai Ninth People's Hospital, Shanghai Jiao Tong University School of Medicine (Approval No. SH9H‐2026‐A1287‐1), and performed in accordance with the guidelines established by the Institutional Animal Care and Use Committee.

### 4.4 Cell Isolation and Culture

BMDMs and BMSCs were freshly isolated from the femurs of 2‐month‐old or 18‐month‐old male C57BL/6 mice. BMDMs were cultured in high‐glucose Dulbecco's modified Eagle medium (DMEM) supplemented with 10% fetal bovine serum (FBS) (Gibco, USA), 1% penicillin‐streptomycin (Gibco, USA), and 20 ng/mL macrophage colony‐stimulating factor (M‐CSF) (TargetMol, China). BMSCs were cultured in α‐minimum essential medium (α‐MEM) supplemented with 10% FBS and 1% penicillin‐streptomycin. RAW 264.7 cells were maintained in high‐glucose DMEM containing 10% FBS and 100 U/mL penicillin‐streptomycin. All cells were cultured in a humidified incubator at 37°C with 5% CO_2_. Sn‐BMDMs were treated with 20 µM quercetin (MCE, USA) and 200 nM dasatinib (MCE, USA) for 24 h to induce senescent cell clearance [3]. To establish the oxidative stress‐induced senescence model, RAW 264.7 cells were treated with 300 µM H_2_O_2_ for 24 h. For macrophage polarization, BMDMs differentiated for 7 days were stimulated with lipopolysaccharide (LPS, 100 ng/mL) and interferon‐γ (IFN‐γ, 50 ng/mL) to induce the M1 phenotype, or with interleukin‐4 (IL‐4, 10 ng/mL) to induce the M2 phenotype. Cells were additionally treated with nHAP or Li‐nHAP at 2 µg/mL. For the LiCl group, Li^+^ concentration was quantified by ICP‐OES corresponding to the concentration released from 5%Li‐nHAP at day 21.

### 4.5 Conditioned Medium (CM) Preparation and In Vitro Osteogenic Differentiation Assay

To obtain macrophage CM, BMDMs subjected to various treatments were washed twice with PBS and cultured in fresh complete medium for 2 days. The supernatant was collected, centrifuged at 2,000 × g for 10 min to remove debris, and stored at −80°C. For CM‐based osteogenic differentiation assays, young or senescent BMSCs were seeded in well plates and cultured until approximately 80% confluence. The medium was then replaced with a 1:1 mixture of the BMDMs‐CM and standard osteogenic medium supplemented with 50 µg/mL ascorbic acid, 10 mM β‐glycerophosphate, and 10 nM dexamethasone (Sigma, Germany). The induction medium was refreshed every 2–3 days. After 10 days of osteogenic induction, BMSCs were fixed with 4% paraformaldehyde (PFA) for 20 min and subjected to alkaline phosphatase (ALP) staining using a BCIP/NBT Alkaline Phosphatase Color Development Kit (Beyotime, China). After 21 days of osteogenic induction, calcium deposition was visualized by Alizarin Red S staining (Oricell, China). ALP and ARS staining were quantified using ImageJ software (National Institutes of Health, USA).

### 4.6 Cell Viability Assay

Cell viability was assessed using a Cell Counting Kit‐8 (Beyotime, China). RAW 264.7 cells or BMDMs were seeded in 96‐well plates at a density of 5000‐10000 cells per well in 100 µL of culture medium. After 24 h of attachment, cells were treated with 0.5, 1, 2, 4, 8 µg/mL of nHAP, 5%Li‐nHAP, or 10%Li‐nHAP for 1, 3, or 5 days. Before each CCK‐8 measurement, the medium was removed and replaced with 100 µL of fresh culture medium to minimize potential interference of nanomaterial with absorbance measurements. Then 10 µL of CCK‐8 reagent was added to each well containing 100 µL of fresh medium. After 1 h of incubation, the absorbance was measured at 450 nm by a microplate reader (Bio‐TEK, USA).

### 4.7 Reverse Transcription‐Quantitative PCR (RT‐qPCR)

RAW 264.7 cells, BMDMs, or BMSCs were harvested for RT‐qPCR analysis after different treatments. Total RNA was extracted using an RNA extraction kit (Fastagen, China). After quality assessment, 1 µg of total RNA was reverse‐transcribed into cDNA using PrimeScript RT Master Mix (Takara, Japan). Quantitative PCR was then performed using TB Green Premix Ex Taq (Takara, Japan). Relative gene expression was calculated using the comparative cycle threshold (2^−ΔΔCt^) method and normalized to *Gapdh*. All primer sequences are listed in Table S1.

### 4.8 Western Blotting

Cells were lysed in X‐RIPA UltraMix (X‐Blot, China). Lysates were centrifuged at 12,000 × g for 10 min at 4°C, and the supernatants were collected. Protein concentration was determined using a bicinchoninic acid (BCA) protein assay kit (Beyotime, China). Equal amounts of protein were separated by 4–20% SurePAGE gels (GenScript, China) and transferred onto 0.22 µm polyvinylidene fluoride (PVDF) membranes (Millipore, USA). Membranes were blocked with X‐Blocking (X‐Blot, China) for 10 min at room temperature and then incubated overnight at 4°C with primary antibodies against p21 (1:1000, 28248‐1‐AP, Proteintech, China), p53 (1:1000, 2524T, CST, USA), GPX4 (1:1000, T56959, Abmart, China) and GAPDH (1:5000, 10494‐1‐AP, Proteintech, China). After washing, membranes were incubated with HRP‐conjugated goat anti‐mouse IgG (1:8000, SA00001‐1, Proteintech, China) or HRP‐conjugated goat anti‐rabbit IgG (1:10000, SA00001‐2, Proteintech, China) secondary antibodies for 1 h at room temperature. Protein bands were visualized using BeyoECL Moon reagent (Beyotime, China) and detected with a Bio‐Rad chemiluminescence imaging system. GAPDH was used as the control, and band intensities were quantified using ImageJ software.

### 4.9 Immunofluorescence Assay

Cells were fixed in 4% PFA for 10 min, permeabilized with 0.25% Triton X‐100 (Beyotime, China), and blocked with 3% bovine serum albumin (BSA). Cells were then incubated with primary antibodies against OPN (1:300, 32601‐1‐AP, Proteintech, China), γ‐H2AX (1:250, ab81299, Abcam, USA), or ATP6V1A (1:250, ab199326, Abcam, USA) followed by CoraLite488‐conjugated goat anti‐rabbit IgG (H+L) (1:250, SA00013‐2, Proteintech, China) or CoraLite594‐conjugated goat anti‐rabbit IgG (H+L) (1:250, SA00013‐4, Proteintech, China). Nuclei were counterstained with DAPI (Beyotime, China) for 5 min. Fluorescence images were acquired using Leica DMi8 fluorescence microscope (Leica, Germany) or Nikon A1 confocal microscope (Nikon, Japan). Colocalization was quantified using Pearson's correlation coefficient in ImageJ software.

### 4.10 Assessment of Ferroptosis‐Associated Parameters

Lipid peroxidation was detected using the Lipid Peroxidation Assay Kit with BODIPY 581/591 C11 (Beyotime, China). Intracellular labile Fe^2^^+^ levels were determined using the Cellular Red Fluorescent Ferrous Ion Assay Kit (Beyotime, China), followed by quantitative analysis via fluorescence microscopy or flow cytometry. Malondialdehyde (MDA) levels were measured by the Lipid Peroxidation MDA Assay Kit (Beyotime, China). Glutathione (GSH) content was assessed using the GSH and GSSG Assay Kit (Beyotime, China). Intracellular ROS levels were examined using the Reactive Oxygen Species Assay Kit (Beyotime, China). Total intracellular iron in BMDMs was quantified using a Ferric and Ferrous Ion Assay Kit (Beyotime, China). To reactivate ferroptosis, Sn‐BMDMs were treated with 5 µµ Erastin (TargetMol, China) for 24 h. All procedures were performed according to the manufacturer's instructions.

### 4.11 Enzyme‐Linked Immunosorbent Assay (ELISA)

The culture supernatants of BMDMs or RAW264.7 were collected, and the levels of SASP factors were determined using mouse TNF‐α and MMP3 ELISA kits (Elabscience, China) in accordance with the manufacturer's instructions.

### 4.12 Senescence‐Associated β‐Galactosidase Staining

β‐Galactosidase activity was assessed using a β‐galactosidase staining kit (Solarbio, China) according to the manufacturer's instructions. Briefly, cells were fixed and incubated with the staining solution at 37°C in a CO_2_‐free incubator. After 24 h, images were captured, and the percentage of SA‐β‐gal‐positive cells was quantified.

### 4.13 Assessment of Lysosomal Homeostasis

Lysosomal acidity was assessed based on relative fluorescence intensity using LysoSensor Green DND‐189 (Thermo Fisher, USA). Lysosomal membrane permeability was assessed using an Acridine Orange Staining Kit (Beyotime, China). Cathepsin B (CTSB) activity was determined using the Cathepsin B Activity Fluorometric Assay Kit (Elabscience, China). DQ‐BSA Green (ShareBio, China) was used to assess lysosomal proteolytic activity. Lysosomes were stained with LAMP1 (1:400, 65050‐1‐Ig, Proteintech, China) for localization. Intralysosomal labile Fe^2^^+^ levels were assessed using Lyso‐FerroRed (Dojindo, Japan). Nuclei were counterstained with Hoechst 33342 (Beyotime, China). 40 nM Bafilomycin A1 (BafA1; TargetMol, China) was used to inhibit lysosomal acidification for 24 h. 500 µM L‐Leucyl‐L‐leucine methyl ester hydrochloride (LLOME; TargetMol, China) was applied for 2 h to induce lysosomal membrane permeabilization. All procedures were performed according to the manufacturer's instructions.

### 4.14 In Vivo Mandibular Bone Defect Model and Material Implantation

A 2.3 mm full‐thickness mandibular defect was selected based on our previously established critical‐size defect model in C57BL/6 mice, where defects of this size showed minimal spontaneous bone healing and remained ununited at 8 weeks [70]. Briefly, 18‐month‐old male C57BL/6 mice were anesthetized. The mandibular region was shaved and disinfected, and the masseter muscle was incised to expose the bone surface. A full‐thickness circular defect with a diameter of 2.3 mm was created at the central region of the mandibular body using a high‐speed bur. The mice were randomly assigned to four groups followed by photocuring and suturing. After 8 weeks, all mice were euthanized, and mandibles, hearts, livers, spleens, lungs, kidneys, and plasma samples were collected for subsequent analyses. An additional cohort of aged mice was subjected to the same four‐group treatment and maintained for 12 weeks post‐surgery to evaluate the durability of bone regeneration. Mandibular specimens were collected at 12 weeks for Micro‐CT and histological analyses. For in vivo macrophage depletion, macrophages were depleted using a Clodronate Liposomes Kit (Yeasen Biotechnology, China). Mice received an initial dose of 200 µL clodronate liposomes per mouse via tail‐vein injection, followed by 200 µL every 3 days; control animals received an equivalent volume of control liposomes according to the same schedule.

### 4.15 Bulk RNA Sequencing and Analysis

Total RNA was extracted with TRIzol reagent (Invitrogen, USA), and samples with RIN values > 7.0 were used for library construction. Poly(A) RNA was enriched, fragmented, reverse‐transcribed into cDNA, and sequenced on the Illumina NovaSeq 6000 platform in 2 × 150 bp paired‐end mode. Raw reads were filtered to remove low‐quality reads and aligned to the reference genome using HISAT2. StringTie was used for transcript assembly and gene expression quantification, and edgeR was applied to identify differentially expressed genes based on raw read counts.

### 4.16 Data‐Independent Acquisition (DIA) Proteomics and Analysis

Proteins were extracted, reduced, alkylated, and digested overnight with trypsin. Peptides were desalted and reconstituted in 0.1% formic acid. LC‐MS/MS analysis was performed in DIA mode using an EASY‐nLC 1200 system coupled to an Orbitrap Exploris 480 mass spectrometer with a 120 min gradient and a Vanquish Neo UHPLC system coupled to an Orbitrap Astral mass spectrometer with an 8 min gradient. DIA data were analyzed using DIA‐NN against the UniProt database with trypsin specificity, allowing up to two missed cleavages. Carbamidomethylation of cysteine was set as a fixed modification, whereas oxidation of methionine and N‐terminal acetylation were set as variable modifications. The results were filtered at a 1% FDR at both peptide and protein levels.

### 4.17 Untargeted Metabolomics and Analysis

Metabolites were extracted with 80% methanol, and the supernatants were collected for UPLC‐HRMS analysis after centrifugation. Pooled QC samples were prepared by mixing equal aliquots from all samples. Metabolite separation was performed on an ACQUITY UPLC HSS T3 column using gradient elution, and metabolites were detected on an Orbitrap Exploris 120 mass spectrometer in both positive and negative ion modes with DDA acquisition. Raw data were processed using XCMS, and metabolites were annotated by matching against the KEGG and HMDB databases with a mass tolerance of <10 ppm. Data were filtered, imputed, normalized, and statistically analyzed using R to identify differentially abundant metabolites.

### 4.18 Micro‐CT and Histological Examination

At the endpoints of 8 or 12 weeks post‐implantation, C57BL/6 mice were humanely euthanized, and their mandibles were excised and subjected to fixation in 4% PFA. Subsequent Micro‐CT scanning of the mandibles was conducted utilizing the SkyScan 1076 system (Belgium) at a spatial resolution of 24 µm. For the purpose of 3D reconstruction, gray scale settings were adjusted within the range of 120 to 255. Parameters including bone volume/total volume (BV/TV), trabecular number (Tb.N) and trabecular thickness (Tb.Th) were computed. Following Micro‐CT scanning, the mandibular specimens were decalcified in an EDTA‐based rapid decalcifying solution at 37°C for 1 week, dehydrated, embedded in paraffin. For histological evaluation, specimens were sectioned to a thickness of 4 µm, followed by processing for standard H&E staining and Masson's trichrome staining. Immunohistochemical and immunofluorescence analyses were performed after employing heat‐mediated antigen retrieval using sodium citrate buffer (pH = 6, ab208572, Abcam, USA) for a duration of 20 min. The sections were stained with primary antibodies against OCN (1:250, GB11233, Servicebio, China), BMP2 (1:500, GB15252, Servicebio, China), OPN (1:500, 32601‐1‐AP, Proteintech, China). For dual‐label immunofluorescence, sections were co‐incubated overnight at 4°C with rat antibody against F4/80 (1:100,  71299, CST, USA) and one of the following rabbit antibodies against: p21 (1:250, 28248‐1‐AP, Proteintech, China), γ‐H2AX (1:250, ab81299, Abcam, USA), GPX4 (1:100, T56959, Abmart, China), or 4‐HNE (1:200, A26085, Abclonal, China). After washing, the sections were incubated with secondary antibodies for 1 h at room temperature. Nuclei were counterstained with DAPI for 5 min. Ferric iron deposition was evaluated by Prussian blue staining using a commercial kit (Servicebio, China). Images were acquired using a Leica DMi8 microscope (Leica Microsystems), and quantification was performed with ImageJ.

### 4.19 Statistical Analysis

Statistical analyses were performed using GraphPad Prism 10.1.2 software. Data are presented as mean ± standard deviation (SD). Comparisons between two groups were analyzed using two‐tailed Student's t‐tests or two‐sided Wilcoxon rank‐sum test. For multiple group comparisons, one‐way analysis of variance (ANOVA) was performed, followed by Tukey's post hoc test. Statistical significance was set at ns, not significant; *p < 0.05, **p < 0.01, and ***p < 0.001.

## Author Contributions

**Baixi Chen**: conceptualization, investigation, writing – original draft, methodology, validation, writing – review and editing, software, formal analysis, project administration, visualization, data curation, supervision, resources. **Ziduan Ding**: methodology, supervision, resources, project administration. **Ruomei Li**: methodology, conceptualization, resources. **Cheng Zhu**: methodology, supervision, resources. **Zhiyu Zhu**: methodology, resources. **Zitong Yang**: resources. **Luyao Qu**: resources. **Jin Hao**: conceptualization, investigation, validation, project administration. **Lunguo Xia**: writing – review and editing, project administration, resources, funding acquisition, supervision, validation. **Bing Fang**: validation, funding acquisition, writing – review and editing, project administration, resources, supervision. **Lu Liu**: conceptualization, methodology, funding acquisition, writing – original draft, writing – review and editing, validation, resources, supervision, project administration.

## Funding

This work was supported by National Natural Science Foundation of China (82571126, 82101060, 82301021), National Key Research and Development Program of China (2022YFC2405904), Open Research Projects in Dentistry at the Ninth People's Hospital Affiliated to Shanghai Jiao Tong University School of Medicine (KYQY2022141), Postdoctoral Scientific Research Foundation of Shanghai Ninth People's Hospital, Shanghai Jiao Tong University School of Medicine (Liulu‐202601048).

## Conflicts of Interest

The authors declare no conflicts of interest.

## Supporting information




**Supporting File**: advs78424‐sup‐0001‐SuppMat.docx.

## Data Availability

The public scRNA‐seq dataset analyzed in this study is available from the Gene Expression Omnibus under accession number GSE280914 (https://www.ncbi.nlm.nih.gov/geo/query/acc.cgi?acc=GSE280914). Other data supporting the findings of this study are available from the corresponding author upon reasonable request.

## References

[1] X. He , W. Hu , Y. Zhang , et al., “Cellular Senescence in Skeletal Disease: Mechanisms and Treatment,” Cellular & Molecular Biology Letters 28, no. 1 (2023): 88, 10.1186/s11658-023-00501-5. PMC10612178 37891477

[2] J. N. Farr , M. Xu , M. M. Weivoda , et al., “Targeting Cellular Senescence Prevents Age‐Related Bone Loss in Mice,” Nature Medicine 23, no. 9 (2017): 1072–1079, 10.1038/nm.4385. PMC5657592 28825716

[3] J. Hou , K.‐X. Chen , C. He , et al., “Aged Bone Marrow Macrophages Drive Systemic Aging and Age‐Related Dysfunction via Extracellular Vesicle‐Mediated Induction of Paracrine Senescence,” Nature Aging 4, no. 11 (2024): 1562–1581, 10.1038/s43587-024-00694-0. PMC11564114 39266768

[4] I. A. Salladay‐Perez , I. Avila , L. Estrada , et al., “p21+TREM2+ Senescent Macrophages Fuel Inflammaging and Metabolic Dysfunction‐Associated Steatotic Liver Disease,” Nature Aging 6, no. 4 (2026): 792–815, 10.1038/s43587-026-01101-6. PMC13099426 41991686

[5] D. Clark , S. Brazina , F. Yang , et al., “Age‐Related Changes to Macrophages are Detrimental to Fracture Healing in Mice,” Aging Cell 19, no. 3 (2020): 13112, 10.1111/acel.13112. PMC7059136 32096907

[6] N.‐Y. Zou , R. Liu , M. Huang , et al., “Age‐Related Secretion of Grancalcin by Macrophages Induces Skeletal Stem/Progenitor Cell Senescence During Fracture Healing,” Bone Research 12, no. 1 (2024): 6, 10.1038/s41413-023-00309-1. PMC10808101 38267422

[7] Y. Dong , X. Shen , Q. Zhuang , et al., “Targeting Injury‐Triggered Grancalcin From Senescent Macrophages Enhances Aged Bone Healing,” Acta Biomaterialia 212 (2026): 562–578, 10.1016/j.actbio.2025.12.031. 41412292

[8] J. Yao , Y. Wu , L. Xu , et al., “Targeting CD14 to Inhibit Macrophage Senescence and Regulate the Microenvironment for Enhanced Tendon‐Bone Healing,” The American Journal of Sports Medicine 53, no. 13 (2025): 3192–3206, 10.1177/03635465251383161. 41108053

[9] B. R. Stockwell , “Ferroptosis Turns 10: Emerging Mechanisms, Physiological Functions, and Therapeutic Applications,” Cell 185, no. 14 (2022): 2401–2421, 10.1016/j.cell.2022.06.003. PMC9273022 35803244

[10] J. Zheng and M. Conrad , “Ferroptosis: When Metabolism Meets Cell Death,” Physiological Reviews 105, no. 2 (2025): 651–706, 10.1152/physrev.00031.2024. 39661331

[11] L. Liu , Z. Zheng , W. You , et al., “Vitamin C Inhibits ACSL4 to Alleviate Ferro‐Aging in Primates,” Cell Metabolism 38, no. 4 (2026): 673–693.e17, 10.1016/j.cmet.2026.02.010. 41819088

[12] H.‐J. Fu , X.‐Y. Zhou , D.‐L. Qin , et al., “Inhibition of Ferroptosis Delays Aging and Extends Healthspan across Multiple Species,” Advanced Science 12, no. 22 (2025): 2416559, 10.1002/advs.202416559. PMC12165029 40162524

[13] D. Tang , G. Kroemer , and R. Kang , “Ferroptosis in Immunostimulation and Immunosuppression,” Immunological Reviews 321, no. 1 (2024): 199–210, 10.1111/imr.13235. 37424139

[14] W. Xiang , T. Zhang , B. Li , et al., “Senescent Macrophages Induce Ferroptosis in Skeletal Muscle and Accelerate Osteoarthritis‐Related Muscle Atrophy,” Nature Aging 5, no. 7 (2025): 1295–1316, 10.1038/s43587-025-00907-0. PMC12270917 40579479

[15] Z.‐Q. Du , J.‐B. Xie , S.‐Y. Ji , W. Zhou , and Z.‐S. Tao , “Spermidine Prevents Iron Overload‐Induced Impaired Bone Mass by Activating SIRT1/SOD2 Signaling in Senile Rat Model,” Redox Report 30, no. 1 (2025): 2485666, 10.1080/13510002.2025.2485666. PMC11966988 40173181

[16] R. A. Weber , F. S. Yen , S. P. V. Nicholson , et al., “Maintaining Iron Homeostasis Is the Key Role of Lysosomal Acidity for Cell Proliferation,” Molecular Cell 77, no. 3 (2020): 645–655.e7, 10.1016/j.molcel.2020.01.003. PMC7176020 31983508

[17] F. Rizzollo , S. More , P. Vangheluwe , and P. Agostinis , “The Lysosome as a Master Regulator of Iron Metabolism,” Trends in Biochemical Sciences 46, no. 12 (2021): 960–975, 10.1016/j.tibs.2021.07.003. 34384657

[18] T. Cañeque , L. Baron , S. Müller , et al., “Activation of Lysosomal Iron Triggers Ferroptosis in Cancer,” Nature 642, no. 8067 (2025): 492–500, 10.1038/s41586-025-08974-4. PMC12158755 40335696

[19] T. M. Loo , X. Zhou , Y. Tanaka , et al., “Senescence‐Associated Lysosomal Dysfunction Impairs Cystine Deprivation‐Induced Lipid Peroxidation and Ferroptosis,” Nature Communications 16, no. 1 (2025): 6617, 10.1038/s41467-025-61894-9. PMC12307602 40731111

[20] M. Gao , P. Monian , Q. Pan , W. Zhang , J. Xiang , and X. Jiang , “Ferroptosis Is an Autophagic Cell Death Process,” Cell Research 26, no. 9 (2016): 1021–1032, 10.1038/cr.2016.95. PMC5034113 27514700

[21] S. Ma , E. S. Henson , Y. Chen , and S. B. Gibson , “Ferroptosis Is Induced Following Siramesine and Lapatinib Treatment of Breast Cancer Cells,” Cell Death & Disease 7, no. 7 (2016): 2307, 10.1038/cddis.2016.208. PMC4973350 27441659

[22] H. Tai , Z. Wang , H. Gong , et al., “Autophagy Impairment With Lysosomal and Mitochondrial Dysfunction Is an Important Characteristic of Oxidative Stress‐Induced Senescence,” Autophagy 13, no. 1 (2017): 99–113, 10.1080/15548627.2016.1247143. PMC5240829 27791464

[23] A. J. Stranks , A. L. Hansen , I. Panse , et al., “Autophagy Controls Acquisition of Aging Features in Macrophages,” Journal of Innate Immunity 7, no. 4 (2015): 375–391, 10.1159/000370112. PMC4386145 25764971

[24] Y.‐Y. Li , B. Ma , J.‐W. Lu , et al., “Lysosome‐Featured Cell Aggregate‐Released Extracellular Vesicles Regulate Iron Homeostasis and Alleviate Post‐Irradiation Endothelial Ferroptosis for Mandibular Regeneration,” Advanced Science 12, no. 34 (2025): 05070, 10.1002/advs.202505070. PMC12442703 40546115

[25] H. Wu , Q. Zhang , J. Zhu , L. Wu , Y. Xiao , and X. Yang , “Biomaterials Targeting Senescent Cells for Bone Regeneration: State‐of‐the‐Art and Future Perspectives,” Bioactive Materials 54 (2025): 686–714, 10.1016/j.bioactmat.2025.09.002. PMC12454288 40995109

[26] S. Zhang , H. Cui , J. Wang , et al., “HydroWrap for T2DM‐Related Fractures: A Smart H2S‐Delivery Controller Modulating Macrophage Senescence,” Bioactive Materials 51 (2025): 257–273, 10.1016/j.bioactmat.2025.05.007. PMC12144456 40487244

[27] W. Song , W. Liu , S.‐Y. Li , et al., “Remodeling the Senescent Microenvironment for Promoting Osteoporotic Tendon‐to‐Bone Healing via Synergizing Senolytic Quercetin and Aligned Nanowire‐Structured Hydrogels,” ACS nano 19, no. 19 (2025): 18364–18385, 10.1021/acsnano.5c01332. 40325895

[28] M. Yu , L. Chen , X. Cai , et al., “Endogenous Metal Ion‐Enriched Immunostimulating Biomimetic Scaffold Improves In Situ Bone Regeneration,” Advanced Materials 38, no. 11 (2026): 07071, 10.1002/adma.202507071. 41482707

[29] T. Viel , S. Chinta , A. Rane , M. Chamoli , H. Buck , and J. Andersen , “Microdose Lithium Reduces Cellular Senescence in Human Astrocytes—A Potential Pharmacotherapy for Covid‐19?,” aging 12, no. 11 (2020): 10035–10040, 10.18632/aging.103449. PMC7346079 32534451

[30] Y. Fang , B. Chen , Z. Liu , et al., “Age‐Related GSK3β Overexpression Drives Podocyte Senescence and Glomerular Aging,” Journal of Clinical Investigation 132, no. 4 (2022): 141848, 10.1172/JCI141848. PMC8843754 35166234

[31] K. Vachhani , C. Whyne , Y. Wang , D. M. Burns , and D. Nam , “Low‐Dose Lithium Regimen Enhances Endochondral Fracture Healing in Osteoporotic Rodent Bone,” Journal of Orthopaedic Research 36, no. 6 (2018): 1783–1789, 10.1002/jor.23799. 29106746

[32] S. I. Hamstra , B. D. Roy , P. Tiidus , et al., “Beyond Its Psychiatric Use: The Benefits of Low‐Dose Lithium Supplementation,” Current Neuropharmacology 21 (2023): 891–910, 10.2174/1570159X20666220302151224. PMC10227915 35236261

[33] R. Khairova , R. Pawar , G. Salvadore , et al., “Effects of Lithium on Oxidative Stress Parameters in Healthy Subjects,” Molecular Medicine Reports 5 (2012): 680–682, 10.3892/mmr.2011.732. PMC3289682 22200861

[34] Z. Wu , J. Bai , G. Ge , et al., “Regulating Macrophage Polarization in High Glucose Microenvironment Using Lithium‐Modified Bioglass‐Hydrogel for Diabetic Bone Regeneration,” Advanced Healthcare Materials 11, no. 13 (2022): 2200298, 10.1002/adhm.202200298. 35388979

[35] E. Beurel , S. F. Grieco , and R. S. Jope , “Glycogen Synthase Kinase‐3 (GSK3): Regulation, Actions, and Diseases,” Pharmacology & Therapeutics 148 (2015): 114–131, 10.1016/j.pharmthera.2014.11.016. PMC4340754 25435019

[36] H.‐Y. Pan and M. Valapala , “Regulation of Autophagy by the Glycogen Synthase Kinase‐3 (GSK‐3) Signaling Pathway,” International Journal of Molecular Sciences 23, no. 3 (2022): 1709, 10.3390/ijms23031709. PMC8836041 35163631

[37] M. Hu , X. Kang , Z. Liu , et al., “Senescent‐Like Border‐Associated Macrophages Regulate Cognitive Aging via Migrasome‐Mediated Induction of Paracrine Senescence in Microglia,” Nature Aging 5, no. 10 (2025): 2039–2054, 10.1038/s43587-025-00956-5. 40931113

[38] M. T. Islam , E. Tuday , S. Allen , et al., “Senolytic Drugs, Dasatinib and Quercetin, Attenuate Adipose Tissue Inflammation, and Ameliorate Metabolic Function in Old Age,” Aging Cell 22, no. 2 (2023): 13767, 10.1111/acel.13767. PMC9924942 36637079

[39] E. J. Novais , V. A. Tran , S. N. Johnston , et al., “Long‐Term Treatment With Senolytic Drugs Dasatinib and Quercetin Ameliorates Age‐Dependent Intervertebral Disc Degeneration in Mice,” Nature Communications 12, no. 1 (2021): 5213, 10.1038/s41467-021-25453-2. PMC8417260 34480023

[40] L. C. Hofbauer , F. Lademann , and M. Rauner , “Deconstructing Cellular Senescence in Bone and Beyond,” Journal of Clinical Investigation 133, no. 8 (2023): 169069, 10.1172/JCI169069. PMC10104887 37066877

[41] J. S. King , M. Wan , A. Kim , et al., “Effects of Aging on the Immune and Periosteal Response to Fracture Injury,” Bone 198 (2025): 117524, 10.1016/j.bone.2025.117524. PMC12168153 40381878

[42] J. Su , X. Liang , J. Yang , et al., “Mild Photothermal‐Responsive Hydrogel With H2Se Delivery for Anti‐Infection and Microenvironment Remodeling to Bone Regeneration in Diabetic Osteomyelitis,” Bioactive Materials 64 (2026): 159–179, 10.1016/j.bioactmat.2026.04.041. PMC13147789 42099772

[43] L. Li , F. Gao , H. Zhang , Y. Yu , B. Ma , and S. Ge , “A Protective Growth Factor Delivery Strategy Based on Polyphenol‐Protein Self‐Assembly to Promote Inflammatory Bone Regeneration,” Biomaterials 320 (2025): 123272, 10.1016/j.biomaterials.2025.123272. 40120176

[44] Y. Hao , Y. Zhang , J. Sun , et al., “Ferroptosis Targeted Metal‐Phenolic Nanocomposite Hydrogel for Immune Microenvironment Guided Regeneration of Diabetic Bone Defects,” Biomaterials 333 (2026): 124217, 10.1016/j.biomaterials.2026.124217. 42033991

[45] K. Zhu , B. Zhang , Y. Li , et al., “Multimodal Hybrid Nanozymes With Antioxidant Catalytic and Antibiotic‐Free Antibacterial Activities for Enhanced Multi‐Target Sepsis Therapy,” Cell Reports Medicine 7, no. 1 (2026): 102541, 10.1016/j.xcrm.2025.102541. PMC12866163 41564860

[46] Y. Yin , B.‐M. Tian , X. Li , et al., “Gold Nanoparticles Targeting the Autophagy–Lysosome System to Combat the Inflammation‐Compromised Osteogenic Potential of Periodontal Ligament Stem Cells: From Mechanism to Therapy,” Biomaterials 288 (2022): 121743, 10.1016/j.biomaterials.2022.121743. 36030103

[47] T. Yu , H. Zhang , W. Sun , et al., “Nanoplastics Exposure Accelerates the Progression of Osteoarthritis via Lysosomal Destabilization‐Mediated Pyroptosis,” Journal of Advanced Research, ahead of print, April 18, 2026, 10.1016/j.jare.2026.04.045. 42009103

[48] Z. Zhu , R. Li , P. Mei , et al., “Lithium‐Doped Silicon‐Hydroxyapatite Nanowires Alleviate Early Bone Loss in Periodontitis via Γδ T Cell Modulation,” Chemical Engineering Journal 519 (2025): 165072, 10.1016/j.cej.2025.165072.

[49] Y. Wang , S. Anchipolovsky , P. Bhuiyan , et al., “Lithium Chloride Suppresses Ferroptosis of Induced Pluripotent Stem Cells With ApoE4/E4 From a Sporadic Alzheimer's Disease Patient,” Neurotherapeutics 23, no. 2 (2026): e00860, 10.1016/j.neurot.2026.e00860. PMC12993404 41760425

[50] J. Ma , L. Tang , Y. Tan , et al., “Lithium Carbonate Revitalizes Tumor‐Reactive CD8+ T Cells by Shunting Lactic Acid Into Mitochondria,” Nature Immunology 25, no. 3 (2024): 552–561, 10.1038/s41590-023-01738-0. PMC10907288 38263463

[51] H.‐C. Wu , T.‐W. Wang , J.‐S. Sun , et al., “Development and Characterization of a Bioinspired Bone Matrix With Aligned Nanocrystalline Hydroxyapatite on Collagen Nanofibers,” Materials 9, no. 3 (2016): 198, 10.3390/ma9030198. PMC5456656 28773321

[52] X. Zheng , G. Shen , C. Wang , et al., “Bio‐Inspired Murray Materials for Mass Transfer and Activity,” Nature Communications 8, no. 1 (2017): 14921, 10.1038/ncomms14921. PMC5384213 28382972

[53] R. F. McKnight , M. Adida , K. Budge , S. Stockton , G. M. Goodwin , and J. R. Geddes , “Lithium Toxicity Profile: A Systematic Review and Meta‐Analysis,” The Lancet 379, no. 9817 (2012): 721–728, 10.1016/S0140-6736(11)61516-X. 22265699

[54] L. Aron , Z. K. Ngian , C. Qiu , et al., “Lithium Deficiency and the Onset of Alzheimer's Disease,” Nature 645, no. 8081 (2025): 712–721, 10.1038/s41586-025-09335-x. PMC12443616 40770094

[55] M. Giorgio , M. Trinei , E. Migliaccio , and P. G. Pelicci , “Hydrogen Peroxide: A Metabolic by‐Product or a Common Mediator of Ageing Signals?,” Nature Reviews Molecular Cell Biology 8, no. 9 (2007): 722–728, 10.1038/nrm2240. 17700625

[56] S. J. Dixon and J. A. Olzmann , “The Cell Biology of Ferroptosis,” Nature Reviews Molecular Cell Biology 25, no. 6 (2024): 424–442, 10.1038/s41580-024-00703-5. PMC12187608 38366038

[57] J. Liu , D. Tang , and R. Kang , “Targeting GPX4 in Ferroptosis and Cancer: Chemical Strategies and Challenges,” Trends in Pharmacological Sciences 45, no. 8 (2024): 666–670, 10.1016/j.tips.2024.05.006. 38866667

[58] K. Du , L. Wang , J. H. Jun , et al., “Aging Promotes Metabolic Dysfunction‐Associated Steatotic Liver Disease by Inducing Ferroptotic Stress,” Nature Aging 4, no. 7 (2024): 949–968, 10.1038/s43587-024-00652-w. PMC12810195 38918603

[59] B. Galy , M. Conrad , and M. Muckenthaler , “Mechanisms Controlling Cellular and Systemic Iron Homeostasis,” Nature Reviews Molecular Cell Biology 25, no. 2 (2024): 133–155, 10.1038/s41580-023-00648-1. 37783783

[60] S. Lee , N. Hwang , B. G. Seok , S. Lee , S.‐J. Lee , and S. W. Chung , “Autophagy Mediates an Amplification Loop During Ferroptosis,” Cell Death & Disease 14, no. 7 (2023): 464, 10.1038/s41419-023-05978-8. PMC10368698 37491375

[61] P. Gross , “Mitophagy Protects Against Ferroptosis,” Nature Cell Biology 26, no. 9 (2024): 1374, 10.1038/s41556-024-01507-7. 39266699

[62] X. Liu , Z. Zhao , Z. Bian , et al., “Endocytosis Is Essential for Cysteine‐Deprivation‐Induced Ferroptosis,” Molecular Cell 85, no. 17 (2025): 3333–3342.e4, 10.1016/j.molcel.2025.08.006. PMC12416143 40858109

[63] K. Mazan‐Mamczarz , D. Tsitsipatis , B. G. Childs , et al., “Single‐Cell and Spatial Transcriptomics Map Senescent Vascular Cells in Arterial Remodeling During Atherosclerosis in Mice,” Nature Aging 5, no. 8 (2025): 1528–1547, 10.1038/s43587-025-00889-z. PMC12323397 40660002

[64] A. Cheng , K.‐H. Tse , H.‐M. Chow , et al., “ATM Loss Disrupts the Autophagy‐Lysosomal Pathway,” Autophagy 17, no. 8 (2021): 1998–2010, 10.1080/15548627.2020.1805860. PMC8386711 32757690

[65] A. Esposito , S. Pepe , M. S. Cerullo , et al., “ATP6V1A Is Required for Synaptic Rearrangements and Plasticity in Murine Hippocampal Neurons,” Acta Physiologica 240, no. 8 (2024): 14186, 10.1111/apha.14186. 38837572

[66] R. Chen , J. Zou , J. Liu , R. Kang , and D. Tang , “DAMPs in the Immunogenicity of Cell Death,” Molecular Cell 85, no. 20 (2025): 3874–3889, 10.1016/j.molcel.2025.09.007. 41106375

[67] K. N. Fountoulakis , M. Tohen , and C. A. Zarate , “Lithium Treatment of Bipolar Disorder in Adults: A Systematic Review of Randomized Trials and Meta‐Analyses,” European Neuropsychopharmacology 54 (2022): 100–115, 10.1016/j.euroneuro.2021.10.003. PMC8808297 34980362

[68] A. G. Kurian , R. K. Singh , K. D. Patel , J.‐H. Lee , and H.‐W. Kim , “Multifunctional GelMA Platforms With Nanomaterials for Advanced Tissue Therapeutics,” Bioactive Materials 8 (2022): 267–295, 10.1016/j.bioactmat.2021.06.027. PMC8424393 34541401

[69] K. Yue , G. Trujillo‐de Santiago , M. Moisés Alvarez , A. Tamayol , N. Annabi , and A. Khademhosseini , “Synthesis, Properties, and Biomedical Applications of Gelatin Methacryloyl (GelMA) Hydrogels,” Biomaterials 73 (2015): 254–271, 10.1016/j.biomaterials.2015.08.045. PMC4610009 26414409

[70] F. Yu , L. Liu , L. Xia , and B. Fang , “Establishment of a C57BL/6 Mandibular Critical‐Size Bone Defect Model,” Journal of Craniofacial Surgery 32, no. 7 (2021): 2562–2565, 10.1097/SCS.0000000000007798. 34054086

[71] Y. Xu , K. Ma , L. Zhang , and G. Li , “Supercharging Cancer‐Fighting T Cells With Lithium Carbonate,” Cell Metabolism 36, no. 3 (2024): 463–465, 10.1016/j.cmet.2024.02.006. 38447529

[72] S. Guo , K. Arai , M. F. Stins , D.‐M. Chuang , and E. H. Lo , “Lithium Upregulates Vascular Endothelial Growth Factor in Brain Endothelial Cells and Astrocytes,” Stroke; A Journal of Cerebral Circulation 40, no. 2 (2009): 652–655, 10.1161/STROKEAHA.108.524504. PMC3691992 18974377

[73] C. Pan , L. Chen , R. Wu , et al., “Lithium‐Containing Biomaterials Inhibit Osteoclastogenesis of Macrophages in Vitro and Osteolysis in Vivo,” Journal of Materials Chemistry B 6, no. 48 (2018): 8115–8126, 10.1039/c8tb02678e. 32254931

[74] T. W. Andrew , L. S. Koepke , Y. Wang , et al., “Sexually Dimorphic Estrogen Sensing in Skeletal Stem Cells Controls Skeletal Regeneration,” Nature Communications 13, no. 1 (2022): 6491, 10.1038/s41467-022-34063-5. PMC9618571 36310174

[75] K. S. Forsyth , N. Jiwrajka , C. D. Lovell , N. E. Toothacre , and M. C. Anguera , “The conneXion Between Sex and Immune Responses,” Nature Reviews Immunology 24, no. 7 (2024): 487–502, 10.1038/s41577-024-00996-9. PMC11216897 38383754

