# Supplementary material for: Lithium‐Doped Hydroxyapatite Nanowires Rejuvenate Senescent Macrophages Via the Lysosome‐Dependent Ferroptosis Axis to Promote Aged Bone Regeneration
Source: Adv Sci (Weinh). 2026 Oct 25:e78424. Online ahead of print. doi: 10.1002/advs.78424 (PMC13696786; doi:10.1002/advs.78424)
Supplement: Supplementary file 1 [file ADVS-9999-e78424-s001.docx]

Supporting Information

**Lithium-Doped Hydroxyapatite Nanowires Rejuvenate Senescent Macrophages via the Lysosome-Dependent Ferroptosis Axis to Promote Aged Bone Regeneration**

Baixi Chen, Ziduan Ding, Ruomei Li, Cheng Zhu, Zhiyu Zhu, Zitong Yang, Luyao Qu, Jin Hao, Lunguo Xia*, Bing Fang*, Lu Liu*

**Figure S1. Conditioned medium from Sn-BMDMs impairs the osteogenic differentiation of BMSCs.**

**(A, F)** Relative mRNA expression of osteogenic genes (*Alpl*, *Spp1*, *Col1a1* and *Runx2*) in young (A) and senescent (F) BMSCs treated with Yg-BMDMs CM, Sn-BMDMs CM, or Sn-BMDMs +D&Q CM. n = 4.

**(B-E, G-J)** ALP and ARS staining in young (B, C) and senescent (G, H) BMSCs, with corresponding quantitative analyses (D, E, I, J). n = 3. Data are presented as mean ± SD. Statistical significance was determined by one-way ANOVA followed by Tukey's post hoc test. *p < 0.05, **p < 0.01, and ***p < 0.001.

**Figure S2. Quality control and profiling validation for the multi‑omics analyses.**

**(A, B)** PCA plot (A) and volcano plot (B) of transcriptomic data. n = 6. **(C, D)** PCA plot (C) and volcano plot (D) of proteomic data. n = 4. **(E, F)** PLS‑DA plot (E) and volcano plot (F) of untargeted metabolomic data. n = 6.

**Figure S3. *In vivo* biocompatibility of the nanowires.**

**(A–C)** CCK‑8 assay of RAW 264.7 cells treated with different concentrations of nHAP (A), 5%Li‑nHAP (B), or 10%Li‑nHAP (C) on days 1, 3, and 5. n = 4. **(D)** CCK‑8 assay of BMDMs. n = 4. **(E, F)** Representative photographs (E) and quantitative analysis (F) of the hemolysis assay for erythrocytes. Triton X-100 and PBS served as the positive and negative controls. n = 3. Data are presented as mean ± SD. Significance annotations in the figure indicate comparisons with the Control group. Statistical significance was determined by one-way ANOVA at each time point followed by Tukey's post hoc test. ns, not significant; *p < 0.05, **p < 0.01, and ***p < 0.001.

**Figure S4. Transcriptomic and metabolomic profiling of the anti-senescence effects of Li-nHAP.**

(A) KEGG enrichment analysis comparing 5%Li‑nHAP‑treated Sn‑BMDMs with nHAP‑treated group. (B) Heatmap of senescence‑inducing and SASP‑related gene expression in BMDMs. (C-F) GSEA of DNA replication (C), mismatch repair (D), cell cycle (E), and PPAR signaling pathway (F) comparing 5%Li-nHAP-treated Sn-BMDMs with Sn-BMDMs. (G) Combined transcriptomic and metabolomic enrichment analysis comparing 5%Li‑nHAP‑treated Sn‑BMDMs with Sn‑BMDMs.

**Figure S5. Li-nHAP modulates macrophage polarization toward an anti-inflammatory phenotype.**
(A–C) Relative mRNA expression of the M1-associated genes *Nos2* (A), *Tnf* (B), and *Il1b* (C) in BMDMs. (D, E) Relative mRNA expression of the M2-associated genes *Arg1* (D) and *Il10* (E) in BMDMs. n = 4. Data are presented as mean ± SD. Statistical significance was determined by one-way ANOVA followed by Tukey’s post hoc test. ns, not significant; *p < 0.05 and ***p < 0.001.

**Figure S6. Li‑nHAP exerts negligible effects on mitochondrial iron metabolism and mitophagy.**

**(A-F)** RT-qPCR detection of mitophagy-associated markers (A-D) and mitochondrial iron metabolism/ROS-related genes (E, F) in BMDMs. n = 4. Data are presented as mean ± SD. Statistical significance was determined by one-way ANOVA followed by Tukey's post hoc test. ns, not significant.

**Figure S7. IPA reveals altered lysosomal iron homeostasis comparing Sn-BMDMs with Yg-BMDMs.**

IPA combined transcriptomic and proteomic data. Red nodes in the figure represent predicted upregulated molecules, whereas blue nodes represent predicted downregulated molecules.

**Figure S8. *In vivo* biosafety evaluation of nHAP+GelMA and Li-nHAP+GelMA.**

**(A)** SEM images of GelMA, nHAP+GelMA, and Li‑nHAP+GelMA. **(B-D)** Hematological parameters, including white blood cell (WBC), red blood cell (RBC), and platelet (PLT) counts. n = 3. **(E, F)** Liver function indicators, including alanine aminotransferase (ALT) and aspartate aminotransferase (AST). n = 3. **(G, H)** Renal function indicators, including blood urea nitrogen (BUN) and creatinine (CR). n = 3. **(I, J)** Plasma levels of lactate dehydrogenase (LDH) and creatine kinase (CK). n = 3. **(K)** Representative H&E staining of major organs (heart, liver, spleen, lung, and kidney) in aged mice. n = 3. Data are presented as mean ± SD. Statistical significance was determined by one-way ANOVA followed by Tukey's post hoc test. ns, not significant.

**Figure S9. Sustained bone regenerative efficacy of Li-nHAP+GelMA at 12 weeks after implantation in aged mice.**

(A) Representative Micro-CT images and 3D reconstructions of mandibular defects in the Blank, GelMA, nHAP+GelMA, and Li-nHAP+GelMA groups at 12 weeks post-surgery. (B–D) Quantitative Micro-CT analysis of bone volume/total volume (BV/TV) (B), trabecular number (Tb.N) (C), and trabecular thickness (Tb.Th) (D). n = 6. (E, G) Representative immunohistochemical staining of OCN (E) and quantitative analysis (G). (F, H) Representative immunohistochemical staining of BMP2 (F) and quantitative analysis (H). n = 6. Data are presented as mean ± SD. Statistical significance was determined by one-way ANOVA followed by Tukey’s post hoc test. ns, not significant; *p < 0.05, **p < 0.01, and ***p < 0.001.

**Figure S10. Macrophage depletion attenuates the regenerative efficacy of Li-nHAP+GelMA in aged mandibular defects.**

(A) Representative immunofluorescence images of F4/80⁺ macrophages within the mandibular defect region. (B) Representative Micro-CT images and 3D reconstructions of mandibular defects at 8 weeks post-surgery. (C–E) Quantitative Micro-CT analysis of bone volume/total volume (BV/TV) (C), trabecular number (Tb.N) (D), and trabecular thickness (Tb.Th) (E). n = 6. Data are presented as mean ± SD. Statistical significance was determined by one-way ANOVA followed by Tukey’s post hoc test. *p < 0.05, **p < 0.01, and ***p < 0.001.

**Figure S11. Pharmacological induction of ferroptosis attenuates the regenerative efficacy of Li-nHAP+GelMA in aged mandibular defects.**

(A) Representative Micro-CT images and 3D reconstructions of mandibular defects at 8 weeks post-surgery. (B–D) Quantitative Micro-CT analysis of bone volume/total volume (BV/TV) (B), trabecular number (Tb.N) (C), and trabecular thickness (Tb.Th) (D). n = 6. Data are presented as mean ± SD. Statistical significance was determined by one-way ANOVA followed by Tukey’s post hoc test. ns, not significant; *p < 0.05, ***p < 0.001.

**Supplementary Table 1. Primers for RT-qPCR analysis.**

| **Genes** | | **Species** | | **Sequence (5′ to 3′)** | |
| --- | --- | --- | --- | --- | --- |
| ***Alpl*** | | **Mus musculus** | | **Forward: CCAACTCTTTTGTGCCAGAGA**  **Reverse: GGCTACATTGGTGTTGAGCTTTT** | |
| ***Runx2*** | | **Mus musculus** | | **Forward: GACTGTGGTTACCGTCATGGC**  **Reverse: ACTTGGTTTTTCATAACAGCGGA** | |
| ***Col1a1*** | | **Mus musculus** | | **Forward: GCTCCTCTTAGGGGCCACT**  **Reverse: ATTGGGGACCCTTAGGCCAT** | |
| ***Spp1*** | | **Mus musculus** | | **Forward: ATCTCACCATTCGGATGAGTCT**  **Reverse: TGTAGGGACGATTGGAGTGAAA** | |
| ***Cdkn2a*** | | **Mus musculus** | | **Forward: CTTCGCCGAGCAGTTTCGT**  **Reverse: TCAATCCCATCAGCCATTTCC** | |
| ***Cdkn1a*** | | **Mus musculus** | | **Forward: GCAGAATAAAAGGTGCCACAGG**  **Reverse: AAAGTTCCACCGTTCTCGGG** | |
| ***Trp53*** | | **Mus musculus** | | **Forward: CTCCGAAGACTGGATGACTGC**  **Reverse: TCCATGCAGTGAGGTGATGG** | |
| ***Mmp3*** | | **Mus musculus** | | **Forward: ACATGGAGACTTTGTCCCTTTTG**  **Reverse: TTGGCTGAGTGGTAGAGTCCC** | |
| ***Mmp13*** | | **Mus musculus** | | **Forward: CTTCTTCTTGTTGAGCTGGACTC**  **Reverse: CTGTGGAGGTCACTGTAGACT** | |
| ***Tnf*** | | **Mus musculus** | | **Forward: CCCTCACACTCAGATCATCTTCT**  **Reverse: GCTACGACGTGGGCTACAG** | |
| ***Serpine1*** | | **Mus musculus** | | **Forward: TTCAGCCCTTGCTTGCCTC**  **Reverse: ACACTTTTACTCCGAAGTCGGT** | |
| ***Ccl2*** | | **Mus musculus** | | **Forward: TTAAAAACCTGGATCGGAACCAA**  **Reverse: GCATTAGCTTCAGATTTACGGGT** | |
| ***Atp6v1a*** | | **Mus musculus** | | **Forward: CTACCCAAAATCCGCGATGAG**  **Reverse: CCATGTCACCTTCCAATCGAA** | |
| ***Atp6v1d*** | | **Mus musculus** | | **Forward: GGCAAAGACCGGATTGAAATCT**  **Reverse: GTCGAAATCGAAGAGTTAAGGCA** | |
| ***Atp6v1f*** | | **Mus musculus** | | **Forward: GCGGGCAGAGGTAAGCTAATC**  **Reverse: TTAGGGTGGCGGTTCTTGTTT** | |
| ***Atp6v1g1*** | | **Mus musculus** | | **Forward: CCCAGGCTGAAATTGAACAGT**  **Reverse: TTCTGGAGGACGGTCATCTTC** | |
| ***Atp6v1g2*** | | **Mus musculus** | | **Forward: GAGGAGGCTCAAATGGAGGTG**  **Reverse: CTGAACCTGCCGTCTTGTG** | |
| ***Atp6v1h*** | | **Mus musculus** | | **Forward: GGATGCTGCTGTCCCAACTAA**  **Reverse: TCTCTTGCTTGTCCTCGGAAC** | |
| ***Atp6v1b1*** | | **Mus musculus** | | **Forward: AAGTTTGCCCAGTATGCTGAG**  **Reverse: GCAGGATGTCCCCTGTGAA** | |
| ***Atp6v0a1*** | | **Mus musculus** | | **Forward: GGACCGACAGAGGAGGATG**  **Reverse: GCCAAAGTCAAACTCTTCTGCG** | |
| ***Atp6v0d1*** | | **Mus musculus** | | **Forward: GCTACTTGGAGGGATTAGTGCG**  **Reverse: GCGGAACTCTACTACCATCTTCT** | |
| ***Atp6v0d2*** | | **Mus musculus** | | **Forward: CAGAGCTGTACTTCAATGTGGAC**  **Reverse: AGGTCTCACACTGCACTAGGT** | |
| ***Atp6v0b*** | | **Mus musculus** | | **Forward: AGTTGCTCTACCTCGGGATCT**  **Reverse: ATGCCACATCAAAGCGAAAGC** | |
| ***Atp6v0e*** | | **Mus musculus** | | **Forward: GCATACCACGGCCTTACTGT**  **Reverse: TGATAACTCCCCGGTTAGGAC** | |
| ***Pink1*** | | **Mus musculus** | | **Forward: TTCTTCCGCCAGTCGGTAG**  **Reverse: CTGCTTCTCCTCGATCAGCC** | |
| ***Sqstm1*** | | **Mus musculus** | | **Forward: AGGATGGGGACTTGGTTGC**  **Reverse: TCACAGATCACATTGGGGTGC** | |
| ***Map1lc3b*** | | **Mus musculus** | | **Forward: GACCGCTGTAAGGAGGTGC**  **Reverse: CTTGACCAACTCGCTCATGTTA** | |
| ***Becn1*** | | **Mus musculus** | | **Forward: ATGGAGGGGTCTAAGGCGTC**  **Reverse: TCCTCTCCTGAGTTAGCCTCT** | |
| ***Slc25a28*** | | **Mus musculus** | | **Forward: AGCATTGCGTGATGTACCCG**  **Reverse: CCTGTTGCTGTGACGTTCA** | |
| ***Dhodh*** | | **Mus musculus** | | **Forward: TCTTCACCTCTTACCTGACAGC**  **Reverse: CATGTTGGAGTCCTGAAACGTA** | |
| ***Gapdh*** | | **Mus musculus** | | **Forward: AGGTCGGTGTGAACGGATTTG**  **Reverse: TGTAGACCATGTAGTTGAGGTCA** | |
| ***Nos2*** | **Mus musculus** | | **Forward: GTTCTCAGCCCAACAATACAAGA**  **Reverse: GTGGACGGGTCGATGTCAC** | |  |
| ***Arg1*** | **Mus musculus** | | **Forward: CTCCAAGCCAAAGTCCTTAGAG**  **Reverse: AGGAGCTGTCATTAGGGACATC** | |  |
| ***Il10*** | **Mus musculus** | | **Forward: GCTCTTACTGACTGGCATGAG**  **Reverse: CGCAGCTCTAGGAGCATGTG** | |  |
| ***Il1b*** | **Mus musculus** | | **Forward: GCAACTGTTCCTGAACTCAACT**  **Reverse: ATCTTTTGGGGTCCGTCAACT** | |  |
